# Replication Fork Reversal after Replication–Transcription Collision

**DOI:** 10.1371/journal.pgen.1002622

**Published:** 2012-04-05

**Authors:** Anne L. De Septenville, Stéphane Duigou, Hasna Boubakri, Bénédicte Michel

**Affiliations:** 1 CNRS, Centre de Génétique Moléculaire, UPR3404, Gif-sur-Yvette, France; 2 Université Paris-Sud, Orsay, France; Agency for Science, Technology, and Research, Singapore

## Abstract

Replication fork arrest is a recognized source of genetic instability, and transcription is one of the most prominent causes of replication impediment. We analyze here the requirement for recombination proteins in *Escherichia coli* when replication–transcription head-on collisions are induced at a specific site by the inversion of a highly expressed ribosomal operon (*rrn*). RecBC is the only recombination protein required for cell viability under these conditions of increased replication-transcription collisions. In its absence, fork breakage occurs at the site of collision, and the resulting linear DNA is not repaired and is slowly degraded by the RecJ exonuclease. Lethal fork breakage is also observed in cells that lack RecA and RecD, *i.e.* when both homologous recombination and the potent exonuclease V activity of the RecBCD complex are inactivated, with a slow degradation of the resulting linear DNA by the combined action of the RecBC helicase and the RecJ exonuclease. The sizes of the major linear fragments indicate that DNA degradation is slowed down by the encounter with another *rrn* operon. The amount of linear DNA decreases nearly two-fold when the Holliday junction resolvase RuvABC is inactivated in *recB*, as well as in *recA recD* mutants, indicating that part of the linear DNA is formed by resolution of a Holliday junction. Our results suggest that replication fork reversal occurs after replication–transcription head-on collision, and we propose that it promotes the action of the accessory replicative helicases that dislodge the obstacle.

## Author Summary

Genomes are duplicated prior to cell division by DNA replication, and in all organisms replication impairment leads to chromosome instability. In bacteria, replication and transcription take place simultaneously, and in eukaryotes house-keeping genes are expressed during the S-phase; consequently, transcription is susceptible to impair replication progression. Here, we increase head-on replication–transcription collisions on the bacterial chromosome by inversion of a ribosomal operon (*rrn*). We show that only one recombination protein is required for growth when the *rrn* genes are highly expressed: the RecBCD complex, an exonuclease/recombinase that promotes degradation and RecA-dependent homologous recombination of linear DNA. In the absence of RecBCD, we observe linear DNA that ends in the collision region. This linear DNA is composed of only the origin-proximal region of the inverted *rrn* operon, indicating that it results from fork breakage. It is partly RuvABC-dependent (*i.e.* produced by the *E. coli* Holliday junction resolvase), indicating that blocked forks are reversed. The linear DNA ends up at the inverted *rrn* locus only if the RecJ exonuclease is inactivated; otherwise it is degraded, with major products ending in other upstream *rrn* operons, indicating that DNA degradation is slowed down by ribosomal operon sequences.

## Introduction

Replication arrest is a recognized source of genetic instability in all organisms. Proteins that protect, process, and restart arrested replication forks have been identified, and in eukaryotes their action is coordinated with the induction of a check-point response to prevent cell cycle progression until replication resumes [1], [2], [3], [4]. Model organisms have been used to amplify specific causes of replication arrest in a controlled way, revealing the existence of dedicated pathways of replication resumption. In bacteria, it has been shown that in spite of the existence of several well-characterized replication-restart machineries capable of reloading a replisome at a replication fork, which depend on the nature of the obstruction, most often arrested replication forks do not simply restart [5]. They are first targeted by various enzymes including accessory replicative helicases and recombination proteins [6], [7], [8]. It thus appears that different causes of replication arrest trigger different responses, and that arrested replication forks are channeled to various pathways depending on the original cause of arrest.

One of the first replication impediments recognized as important is the one created by transcription [9], [10], [11], [12], [13]. Enzymes that facilitate replication across highly transcribed regions have been identified in yeast [14]. In bacteria, replication, transcription and translation occur concomitantly, and replication progresses more than 10 times faster than transcription. Consequently, replication-transcription collisions are predicted to occur quite frequently. Head-on collisions between replication and transcription are more dramatic than co-directional collisions [9], [15], [16], nevertheless, under standard growth conditions replication arrests in highly transcribed regions are frequent enough to turn them into detectable hotspots of replication restart [17].

**Figure 1:**
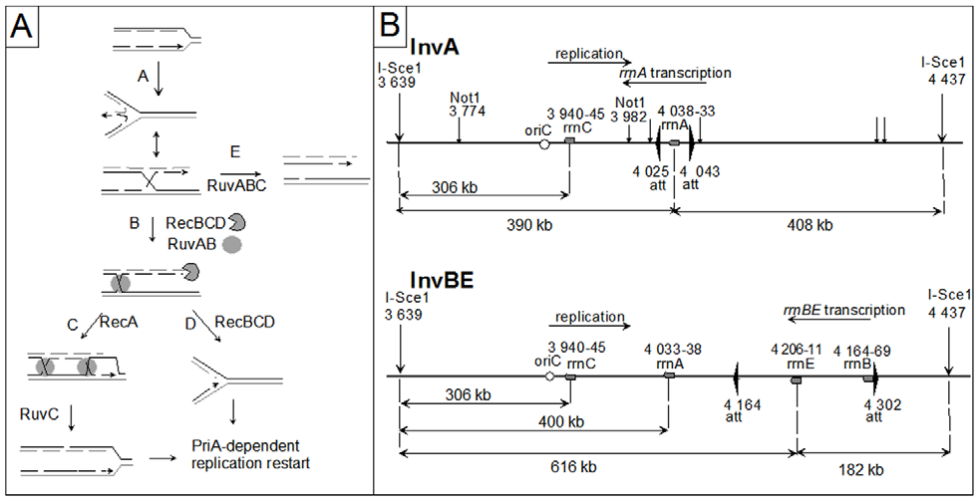
Replication fork reversal model and schematic representation of the I-SceI fragment carrying the inverted region in InvA and InvBE. A Replication fork reversal model. In the first step (A), the replication fork is arrested, and the leading and lagging strand ends of the newly synthesized strands anneal. The reversed fork forms a four-arm structure (Holliday junction, HJ; two alternative representations of this structure are shown, open X and parallel stacked X). RecBC is essential for resetting of the fork, either by RecA-dependent homologous recombination (B–C) or by DNA degradation (B–D). Either pathway creates a substrate for replication restart proteins (PriA and its partners). In the absence of RecBCD (E), resolution of the HJ causes chromosome linearization. Continuous lines: parental chromosome. Dashed lines: newly-synthesized strands. Circle: RuvAB. Incised circle: RecBCD. B Schematic representation of the I-SceI fragment carrying the inverted region in InvA and InvBE. Positions of inversion ends (att) are indicated by wide flat arrowheads. Positions of the cleavage sites (I-*Sce*I, *Not*I) are indicated by vertical arrows. Grey boxes represent the *rrn* genes and the open circle shows the position of *oriC*. The direction of replication and the direction of transcription of the inverted *rrn* are indicated. The distances between the two I-*Sce*I sites and between the I-*Sce*I sites and the 3′ end of the *rrn* operons are shown. The drawing is not to scale.

In *Escherichia coli*, as in other organisms, recombination proteins have been shown to facilitate replication progression under various conditions of replication impairment [6], [18]. In several replication mutants, recombination proteins play a specific role by participating in a reaction named replication fork reversal (RFR) [5], [6], [19], [20], [21]. During RFR, the newly synthesized strands are unwound from the daughter duplexes and base pair to form a Holliday junction (HJ) adjacent to a DNA double-stranded (dsDNA) end. This dsDNA end, made by the annealing of the leading and lagging strand ends, is used to reset a functional replication fork either by RecBCD- RecA- RuvABC- catalyzed homologous recombination (Figure 1A, pathway C), or by RecBCD- catalyzed DNA degradation (Figure 1A, pathway D). RecBC is essential for the viability of replication mutants that undergo RFR, because in its absence the dsDNA end is not efficiently processed, and resolution by RuvABC of the HJ produced by fork reversal results in the formation of a lethal, one-ended, chromosome break (Figure 1A pathway E). Hallmarks of the replication fork reversal reaction are (i) the requirement for viability for RecBC, but not RecA or RuvAB (in contrast with *bona fide* DNA double strand-breaks which requires all these enzymes for repair [22], [23], [24]), and (ii) the observation of RuvABC-dependent fork breakage in the absence of RecBC, while in its presence, fork breakage does not occur [19], [25].

A related reaction was later proposed to promote replication restart after blockage by a RNA polymerase that is itself arrested by a UV lesion [26]. Based on measures of cell survival after UV irradiation in various mutants, it was concluded that replication forks arrested by RNA polymerase in UV irradiated cells were reversed by RecG, an enzyme that binds multiple branched structures *in vivo* and *in vitro* [27]. In contrast with replication mutants, reversed forks in UV-irradiated cells were proposed to be targeted primarily by HJ-binding enzymes, and not by RecBCD: either RecG would convert them back to fork structures and thereby promote restart, or RuvABC would resolve them causing fork breakage. However, this model was later challenged, when direct measures of DNA synthesis showed that the inactivation of *recG* does not prevent replication restart in UV irradiated cells [28] and even promotes it [29], [30]. Furthermore, quantification of UV-induced chromosome fragmentation in *recBC* mutants showed that it completely depends on RuvABC but is hardly influenced by the RecG status of the cell [31].

Interestingly, RNA polymerase is now recognized as the main replication obstacle in the *rep* mutant, where RFR was first described [8], [32]. In *E. coli*, three accessory replicative helicases act at forks blocked by replication-transcription collisions: Rep, UvrD and DinG [8], [33]. Rep is the most critical of the three helicases since it is the only one required for normal replication. In *rep* mutants chromosome replication is twice slower than in wild-type cells [34], probably owing to frequent replication arrest since *rep* mutants undergo RFR and need replication restart proteins for growth [19]. Furthermore, Rep is driven to arrested replication forks by a direct interaction with the replicative helicase DnaB [35]. UvrD can substitute for Rep in its absence, since the *uvrD* single mutant has no replication defect whereas cells that lack both Rep and UvrD have a very low viability [32], [36]. DinG acts as a second back-up; its inactivation is not deleterious in *rep* or *uvrD* single mutants but prevents the residual growth of *rep uvrD* double mutants, as well as the growth of *rep uvrD* mutants in which viability is restored by a suppressor mutation that reduces replication arrest or limits its deleterious consequences (*recF*, *rpoB*, *rpoC* mutations, [8], [32]).

In order to better characterize the action of enzymes recruited to replication-transcription collision sites, we constructed strains in which the inversion of a specific ribosomal operon (*rrn*) creates a strong, locally defined replication obstacle under rapid growth conditions, owing to the high level of *rrn* operon expression (Inv strains, Figure 1B; [8]). This particular experimental setup allowed us to directly demonstrate that *in vivo* Rep, UvrD and DinG helicases indeed act at sites of replication-transcription collisions. Furthermore, in Inv strains the presence of any combination of two of these three accessory replicative helicases is required for growth in rich medium, *i.e.* under high collision conditions, suggesting that two of these helicases act together [8]. In contrast, the transcription-coupled repair factor Mfd does not seem to play a role in the viability of Inv mutants (Table S1, Ref. in Text S1), although this helicase dislodges transcription complexes blocked *in vivo* by a DNA lesion and *in vitro* by various obstacles, including replication forks [37], [38], [39].

In the present work, we used strains carrying one or two inverted *rrn* operons (Inv mutants) to address the question of the role of recombination proteins following replication-transcription collisions. First, we tested the effects of several recombination mutations and combination of mutations on the viability of Inv mutants. We previously reported that Inv strains are not affected by the absence of RecF or RecA, essential for the repair of single-stranded DNA gaps [8]; we identify here the main DSB repair complex RecBC as the only recombination function essential for viability under conditions of high replication-transcription collisions. Second, we looked for the occurrence of DSBs in the region of replication-transcription collisions by direct analysis of chromosomes of different mutants; we actually observed chromosome breakage under conditions of hyper-collisions. Third, we characterized these chromosome breaks and show that only the origin-proximal DNA double-strand end of the transcription-replication collision site can be detected, which is indicative of fork breakage. We also show that linear DNA ends that result from fork breakage are slowly degraded even in the absence of Exo V, the major dsDNA exonuclease; we show that this DNA degradation is mainly RecJ-dependent and is slowed down upon the encounter of other *rrn* loci on the chromosome. Finally, we addressed the question of the enzyme(s) involved in the fork breakage reaction by inactivating candidate genes. Only *ruvAB* inactivation was found to affect the level of fork breakage, indicating a role for the RuvAB complex. Altogether, these results lead us to propose a model for the processing of replication forks arrested by the encounter of an oppositely-oriented highly-transcribed locus.

## Results

### Inv mutants require RecBC for growth on rich medium

**Figure 2:**
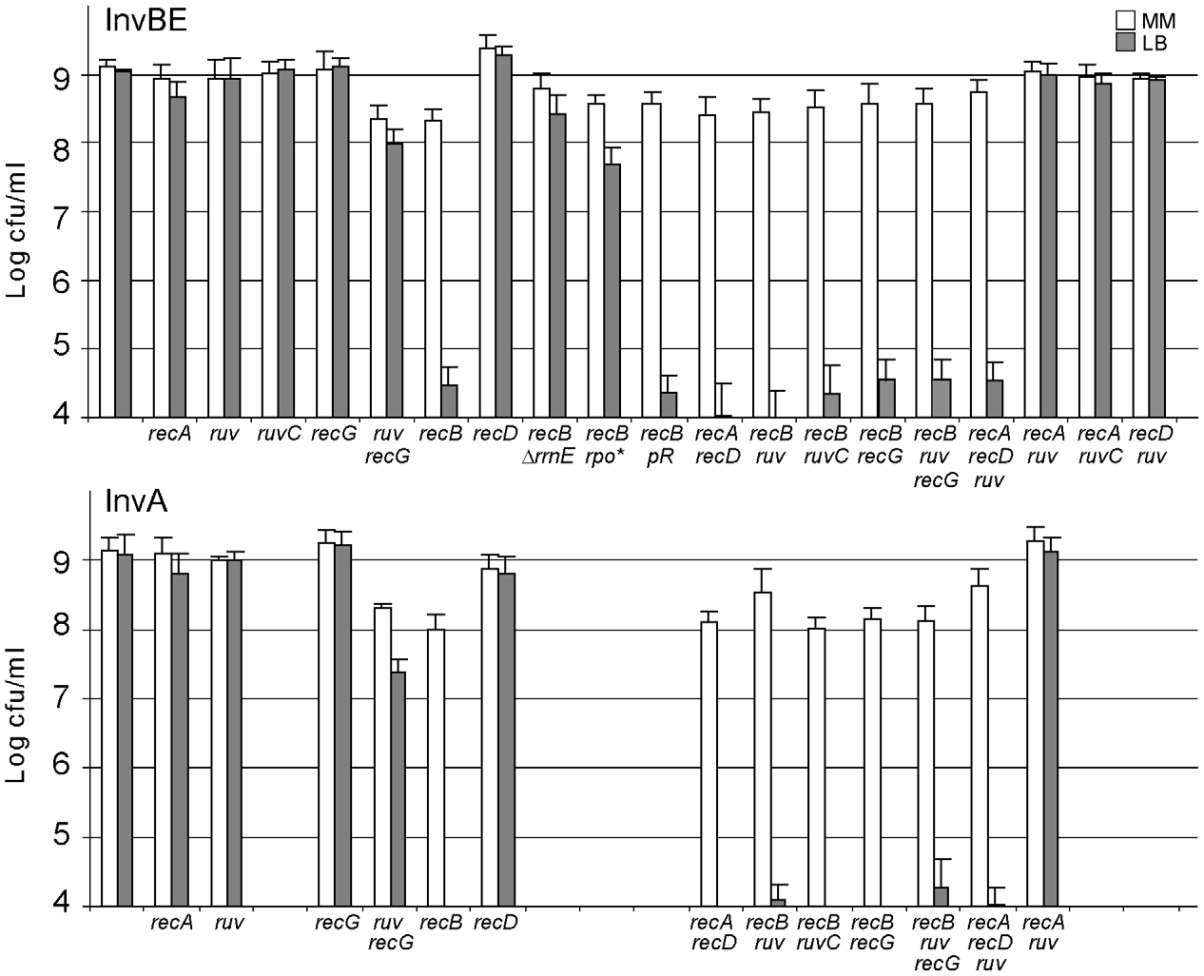
Inv *recB* strains are LB–sensitive. Appropriate dilutions of overnight cultures grown at 37°C in MM (OD 0.8 to 1.5) were plated on MM and LB plates, which were incubated at 37°C. White boxes: colony forming units (cfu)/ml on MM plates after 48 h incubation; grey boxes: cfu/ml on LB plates after 16–24 h incubation. Bars indicate standard deviations. Top: InvBE strains; bottom: InvA strains. *ruv* stands for *ruvAB* inactivation. rpo* stands for the *rpoC* ^Δ215–220^ mutation, pR stands for pEM001, the plasmid encoding RNaseH. Colonies were small in 48 h on MM for Inv *recB ruv* and Inv *recB ruv recG* mutants, but a similar growth delay was observed for non-inverted strains. A small percentage of small colonies was observed after two to four days of incubation on LB with the InvBE *recB ruvAB* (or *recG*) and InvBE *recB ruvAB recG* mutants, however the number of these colonies was highly variable and no colony was ever observed with the InvA *recB ruvAB* (and/or *recG*) mutants, indicating that these mutants still require RecBC for growth on rich medium.

In order to test the role of recombination proteins upon replication-transcription collisions we used the InvA and InvBE strains, which carry a 18 kb inversion encompassing *rrnA*, and a 138 kb inversion containing *rrnB* and *rrnE*, respectively (Figure 1B). *rrn* inversions increase the level of replication-transcription collisions particularly in rich medium (Luria Broth, LB), *i.e.* under conditions of high *rrn* expression and high replication fork density [8]. Recombinational repair of DNA double-stranded breaks (DSBs) requires (i) RecBC, the pre-synaptic protein that loads RecA at dsDNA ends, (ii) RecA, which promotes strand invasion and homology search, and (iii) RuvAB and RuvC (or RecG) which resolve HJs formed by strand exchange [22]. Inverted *rrn* genes do not render homologous recombination essential, as RecA is not required for the growth of Inv strains on LB (Figure 2; [8]). Similarly, we observed that the inactivation of RuvABC and/or RecG, which catalyze the final step of homologous recombination as two redundant pathways of HJ resolution, did not affect the growth of Inv mutants on LB (Inv *ruv*, Inv *recG* and Inv *ruv recG* mutants, Figure 2; the 5-fold reduction of viability of the *ruv recG* combination of mutations in minimal medium was similarly observed in non-inverted strains (data not shown), and the slightly higher reduction of viability in LB might result from an increased need for the resolution of homologous recombination intermediates). However, a *recB* null mutation, which by inactivating the RecBCD complex prevents both the recombinational repair and the degradation of dsDNA ends, strongly decreased the plating efficiency of Inv mutants on LB medium (Figure 2). This observation suggests that dsDNA ends, which need to be acted upon by RecBCD, are formed during replication of the inverted chromosome region. The *recD* mutation, which leaves intact the homologous recombination activity of the RecBC complex but inactivates the efficient dsDNA degradation function of RecBCD, exonuclease V, did not prevent growth of Inv mutants on LB medium, suggesting that in the absence of exo V the recombination function of RecBC (helicase and RecA loading activities) is sufficient for viability.

Although InvBE carries a large inversion, the LB sensitivity of Inv *recB* mutants is due to the inversion of the only highly expressed of the two inverted operons, *rrnE*, since the precise deletion of this operon restored 100% colony formation on LB compared to MM (InvBE Δ*rrnE recB* mutant, Figure 2). It should be noted that the inverted region in the InvBE Δ*rrnE recB* mutant still carries an inverted *rrn* locus, *rrnB*. However, *rrnB* is expressed here at the same level in LB and in MM (minimal medium), owing to a deletion of the promoter enhancer region [8]. The full viability of the InvBE Δ*rrnE recB* mutant shows that RecBC is only needed when the inverted *rrn* operon is highly expressed. In addition, a RNA polymerase mutation that decreases *rrn* operon expression in LB [8], strongly increased the viability of the InvBE *recB* mutant (InvBE *rpoC* ^Δ215–220^ *recB* mutant, Figure 2). Finally, *rrn* genes are prone to the formation of R-loops [40]; however, the requirement for RecBC is not linked to R-loop formation since over-expression of RNaseH, which destroys R-loops, did not restore viability (InvBE *recB* pR; Figure 2). Altogether, these results suggest that the requirement for RecBC is caused by collisions between replication forks and RNA polymerases transcribing the inverted *rrn* operons.

The LB sensitivity of Inv *recB* mutants, together with the lack of LB sensitivity of Inv *recA*, and Inv *ruv recG* mutants, suggest that a dsDNA end is formed upon replication collisions with the inverted *rrn* without actual DNA breakage. These results can be accounted for by the RFR model (Figure 1A). This model predicts that inactivation of both recombination (by a *recA* mutation) and RecBCD-catalyzed degradation (by a *recD* mutation) should be lethal, because it inactivates both pathways of fork resetting. This prediction was tested and, as expected, the combination of a *recA* and a *recD* mutation prevented growth of Inv mutants (InvA *recA recD* and InvBE *recA recD*, Figure 2).

Inactivation of the function catalyzing the first step of fork reversal should restore the viability of Inv *recB* mutants on LB, by precluding the formation of dsDNA ends. RFR has been shown to be catalyzed by various enzymatic activities. Depending on the cause of replication arrest, RecA, RuvAB, and RecG have been implicated in the formation of reversed forks *in vivo* or *in vitro* [6]. The observation that the Inv *recA* mutant is killed by a *recD* mutation indicates that a dsDNA end is still formed in the absence of RecA and that RecA is not the enzyme responsible for fork reversal. As shown in Figure 2, in the absence of either RuvAB or RecG, Inv *recB* and Inv *recA recD* mutants remain lethal on LB, indicating that fork reversal still occurs (Inv *recB ruvAB*, Inv *recD recA ruvAB*, and Inv *recB recG*, Figure 2). Inv *recB* mutants also remained LB sensitive in the absence of both RuvAB and RecG (Inv *recB ruvAB recG*, Figure 2), excluding a redundant function for these two enzymes. Control Inv *recA ruv* and Inv *recD ruv* mutants remained resistant to LB. Therefore, either RFR is not catalyzed in Inv mutants by the RecA, RuvAB or RecG enzymes, or in their absence a redundant, yet unknown enzyme can still catalyze the reaction. High levels of positive super-coiling can promote RFR *in vitro* [41], and could conceivably accumulate at sites of replication-transcription collisions, if the activity of gyrase (the enzyme that removes positive super-coils) was limiting *in vivo*. However, increasing (by inserting a gyrase-specific hotspot sequence at the collision site), or decreasing (by a *gyrBts* mutation) gyrase activity did not affect the viability of any Inv recombination mutant (our unpublished results), which renders unlikely a spontaneous reaction driven by positive super-coiling.

### Fork cleavage at inverted *rrnA* in the InvA *recBC* mutant

**Figure 3:**
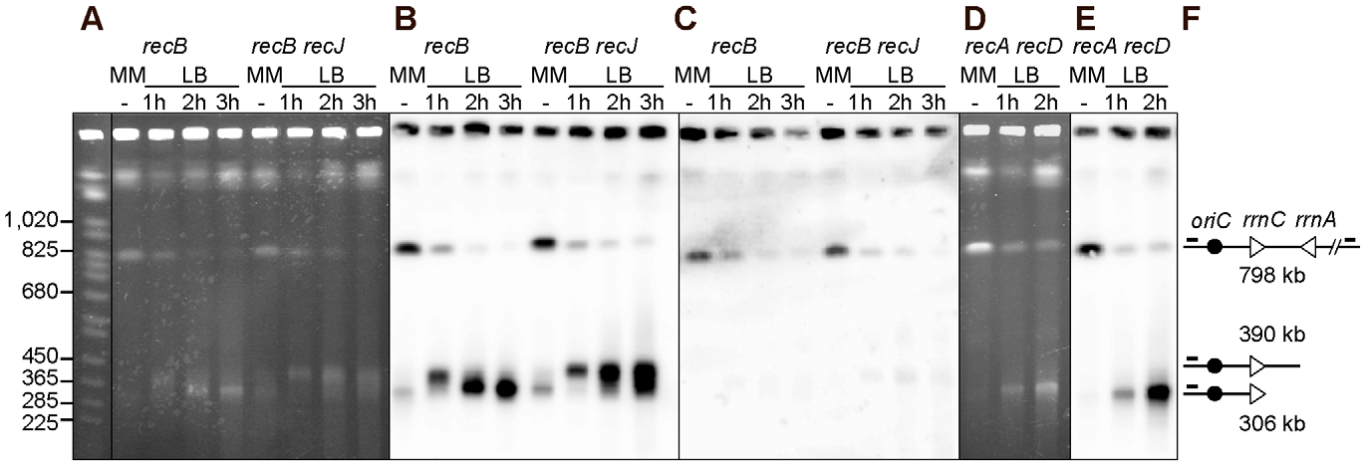
Chromosome breakage in InvA *recB* and InvA *recA recD* mutants. Chromosomes of InvA recombination mutants, grown in minimal medium (MM) or for 1, 2 or 3 hours in LB, were cleaved with the I-*Sce*I enzyme and fragments were separated by PFGE. A. Ethidium bromide stained gel with I-*Sce*I cut chromosomes from InvA *recB* and InvA *recB recJ* mutants. First lane *Saccharomyces cerevisiae* chromosome ladder, relevant sizes are indicated on the left. B. Southern blot of the gel shown in A using the origin-proximal probe in the 800 kb I-*Sce*I fragment, both the intact fragment and fragments of smaller sizes hybridize with the probe. C. Southern blot of the gel shown in A using the origin-distal probe in the 800 kb I-*Sce*I fragment, only the intact I-*Sce*I fragment hybridizes with the probe. D. Ethidium bromide stained gel with I-*Sce*I cut chromosomes from the InvA *recA recD* mutant. E. Southern blot of the gel shown in D using the origin-proximal probe in the 800 kb I-*Sce*I fragment, both the intact fragment and fragments of smaller sizes hybridize with the probe. F. Schematic representation of the different DNA fragments. The triangles represent the two *rrn* operons as indicated, the black circle represent *oriC* and the little bars above the lines represent the position of the origin-proximal (left) and origin distal (right) probes.

According to the RFR model, the dsDNA ends that are formed at blocked forks are converted into one-ended double-strand breaks (DSBs) by the action RuvABC in the absence of RecBC (Figure 1A, pathway E). To ensure that the RecBC requirement results from fork breakage at the inverted *rrn* genes, we performed a molecular analysis of the region of replication-transcription collision. I-*Sce*I sites were introduced into the chromosome on both sides of the inverted regions (Figure 1B). Chromosome breakage at *rrnA* should cleave the ∼800 kb I-*Sce*I fragment into two fragments of about 400 kb, whereas fork breakage is expected to produce only the origin-proximal one of these two fragments (Figure 1B; Figure 1A, pathway E). We analyzed the I-*Sce*I cleavage products of the InvA *recB* chromosome by pulse field gel electrophoresis (PFGE) and Southern blotting, prior to and after a shift to LB, using a probe that hybridizes with the origin-proximal part of the I-*Sce*I fragment carrying the inversion (Figure 3A, 3B). The intact I-*Sce*I fragment observed in MM disappeared after a shift to LB, while a ∼400 kb fragment appeared in 1 hr and seemed to be converted to DNA fragments of smaller size with time, with the accumulation of a fragment of ∼300 kb in three hours (Figure 3A, 3B). The length of the DNA fragment observed after 1 hr of incubation in LB (∼400 kb) corresponds to the distance between the I-*Sce*1 site and the inverted *rrnA* operon (Figure 1B). However, its conversion to smaller fragments at later times suggests that this DNA fragment is degraded *in vivo*. In *E. coli*, one of the main exonucleases besides RecBCD is the RecJ exonuclease, which degrades 5′-ended single-stranded DNA produced by the action on dsDNA ends of RecQ or another helicase [42]. We tested whether the I-*Sce*I - *rrnA* fragment of ∼400 kb was being degraded by RecJ and observed that indeed inactivation of RecJ prevented most of the conversion of this fragment to smaller ones (Figure 3A, 3B). This result shows that RecJ degrades dsDNA ends made by breakage at *rrnA* in the InvA *recB* mutant. No production of linear DNA *in vivo* could be detected with control strains (non-inverted *recB* mutants, InvA RecBCD^+^ strains, Figure S1).

In order to determine whether the origin-distal part of the I-*Sce*I fragment is also produced by cleavage at *rrnA*, the same membranes were hybridized to a probe within this region. The only fragment detected was in the intact I-*Sce*I fragment (Figure 3C), which, as seen with the origin-proximal probe, decreased in intensity with time. As previously observed for other fork-breakage reactions [43], the absence of detectable origin-distal fragment is consistent with the conclusion that the origin-proximal fragments result from fork breakage (one-ended break) and not from a *bona fide* DSB (two-ended break) at *rrnA*.

Our attempts to quantify the bands produced by fork cleavage at *rrnA* provided highly variable results, owing partly to the nearly full disappearance of the intact I-*Sce*I fragment, an unexpected observation, and partly to the fact that a large fraction of the probed DNA remained trapped in the wells. Non-migrating DNA may result from partial cleavage and/or from the presence of structures blocking DNA migration (forks, HJ, replication bubbles), thus contributing to the disappearance of the intact I-*Sce*I fragment upon growth in LB. In order to determine whether the non-migrating DNA is trapped in wells by unresolved recombination intermediates, we analyzed chromosome breakage in the InvA *recA recD* mutant. A large amount of DNA fragments smaller than the intact I-Sce1 fragment was detected in the InvA *recA recD* mutant (Figure 3D, 3E), in contrast with in the non-inverted *recA recD* control strain (Figure S1). The level of non-migrating DNA remained very high and the intact I-*Sce*I fragment disappeared when cells were propagated in LB, still preventing quantification (Figure 3D, 3E). This observation suggests that non-migrating DNA might be trapped in wells owing to the presence of replication intermediate structures. Interestingly, growth of the InvA *recA recD* mutant in LB triggered the appearance of a DNA band that hybridized specifically with the origin proximal probe, and was not the ∼400 kb fragment expected from breakage at *rrnA* but the smaller ∼300 kb one, observed in the *recBC* mutant in the presence of the RecJ exonuclease (Figure 3D, 3E). Since in a *recA recD* context, RecBC and RecJ are active, this ∼300 kb fragment, which is not observed in a *recB recJ* context, is likely to result from the degradation by the combined action of the RecBC helicase and the RecJ exonuclease of a dsDNA end produced by fork cleavage at *rrnA*.

### Fork cleavage at the inverted *rrnE* locus in the InvBE *recBC* mutant

**Figure 4:**
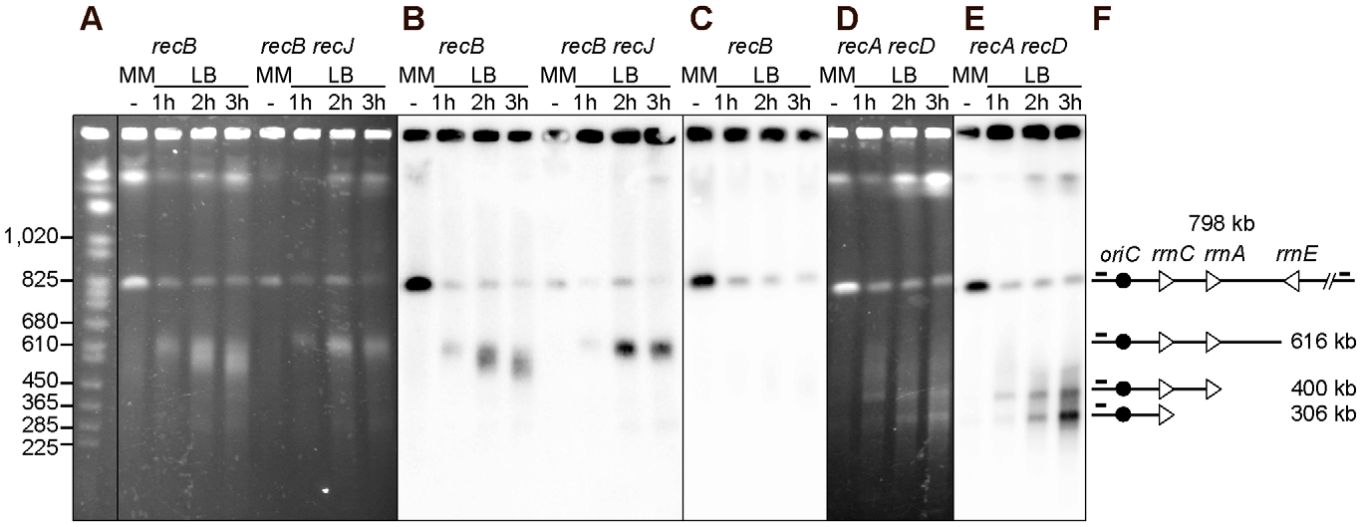
Chromosome breakage in InvBE *recB* and InvA *recA recD* mutants. Chromosomes of the indicated InvBE mutants, grown in minimal medium (MM) or for 1, 2 or 3 hours in LB, were cleaved with the I-*Sce*I enzyme and fragments were separated by PFGE. A. Ethidium bromide stained gel with I-*Sce*I cut chromosomes from InvBE *recB* and InvBE *recB recJ* mutants. First lane *Saccharomyces cerevisiae* chromosome ladder, relevant sizes are indicated on the left. B. Southern blot of the gel shown in A using the origin-proximal probe in the 800 kb I-*Sce*I fragment, both the intact fragment and fragments of smaller sizes hybridize with the probe. C. Southern blot of a gel made with the InvBE *recB* I-*Sce*I cut chromosomes, using the origin-distal probe in the 800 kb I-SceI fragment, only the intact I-*Sce*I fragment hybridizes with the probe. D. Ethidium bromide stained gel with I-*Sce*I cut chromosomes from the InvBE *recA recD* mutant. E. Southern blot of the gel shown in D using the origin-proximal probe in the 800 kb I-*Sce*I fragment, both the intact fragment and fragments of smaller sizes hybridize with the probe. F. Schematic representation of the different DNA fragments. The triangles represent the three *rrn* operons as indicated, the black circle represent *oriC* and the little bars above the lines represent the position of the origin-proximal (left) and origin-distal (right) probes.

To determine whether fork breakage at the inverted *rrnA* locus is specific for this ribosomal operon, we analyzed the formation of linear DNA in the inverted region of the InvBE *recB* mutant by I-*Sce*1 enzyme. When the InvBE *recB recJ* mutant was propagated in LB medium, a fragment of about 616 kb that hybridizes with the origin-proximal probe was observed, as expected from fork breakage at *rrnE* (Figure 4A, 4B). In the InvBE *recB* mutant, this ∼616 kb fragment was transformed into a smear, owing to RecJ-mediated DNA degradation (Figure 4A, 4B). The corresponding origin-distal fragment of about 180 kb was not observed using an origin-distal probe, supporting the conclusion that the origin proximal fragments result from fork breakage and not from a *bona fide* DSB (Figure 4C). In the InvBE *recA recD* mutant, the origin-proximal probe revealed a smear of fragments smaller than 616 kb and two main fragments of ∼400 kb and ∼300 kb (Figure 4D, 4E), which presumably result from degradation of the ∼616 kb fragment by RecBC and RecJ as they are not observed in a InvBE *recB recJ* mutant. As in InvA mutants, the disappearance of the intact I-*Sce*I band and the high level of non-migrating I-*Sce*I fragments prevented the quantification of linear DNA species. No production of linear DNA *in vivo* could be detected with control strains (non-inverted *recB* or *recA recD* mutants, InvBE RecBCD^+^ strains, Figure S1).

### *rrn* genes are a barrier to DNA degradation

**Figure 5:**
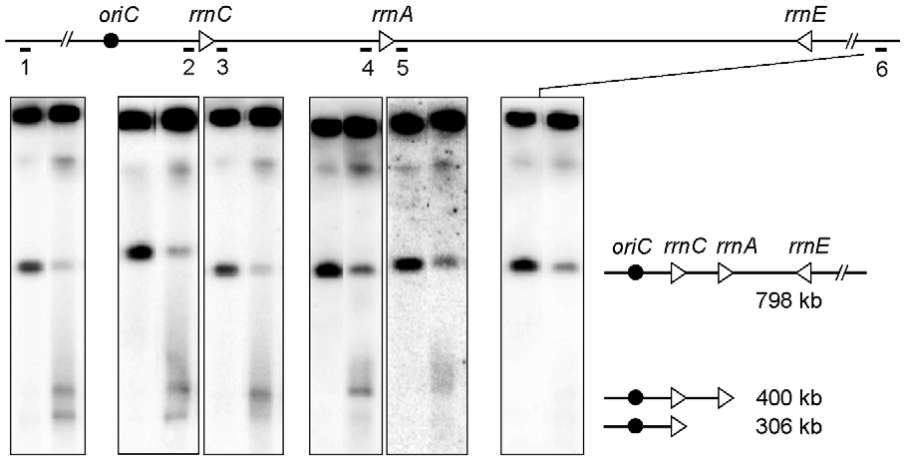
*rrn* operons are a barrier to DNA degradation. Top; schematic representation of the I-*Sce*I fragment carrying the inverted *rrnE* operon. Triangles represent *rrn* operons as indicated, the black circle represents *oriC*, the black line represents the DNA I-*Sce*I fragment and the numbered bars under this line show the positions of the different probes. Bottom; chromosomes of InvBE *recA recD* cells grown in MM or in LB for 2 hours were cleaved with I-*Sce*I and fragments were separated by PFGE, for each panel: left lane, cells grown in MM, right lane, cells grown in LB for 2 hours. Southern blots were hybridized with the different probes indicated above each panel. From left to right: probe 1 - origin-proximal probe, probe 2 - *rrnC* promoter probe, probe 3 - *rrnC* terminator probe, probe 4 - *rrnA* promoter probe, probe 5 - *rrnA* terminator probe, probe 6 - origin-distal probe. A schematic representation of the fragments of different length is shown on the right. For each probe all hybridizing fragments are necessarily larger than the distance between the origin-proximal I-*Sce*I site and the probe, so that the smear stops at the position of the probe.

We noted that the ∼300 kb and the ∼400 kb fragments observed in the InvBE *recA recD* mutant have a size corresponding to the distance between the I-*Sce*I site and the normally oriented *rrnC* and *rrnA* operons, respectively (Figure 1B). We hypothesized that the product of fork breakage at the inverted *rrnE* locus might be degraded until the first non-inverted *rrn* is encountered by exonucleases. This DNA degradation is not observed in a *recB recJ* mutant, is slow in a *recB* mutant where it is mainly catalyzed by RecJ (it does not reach *rrnA*, 231 kb away, in two hours), and is more efficient in a *recA recD* mutant, where the helicase RecBC and RecJ exonuclease are both active (Figure 4). To test the hypothesis that *rrn* genes could be a barrier to DNA degradation, membranes such as the one shown in Figure 4E were hybridized with probes located about 5 kb apart, immediately upstream or downstream of *rrnC* and *rrnA* (Figure 5, only the MM and LB 2 hours lanes are shown, the kinetics of appearance of bands is shown Figure 4E). The ∼300 kb fragment hybridized with the *rrnC* promoter region but not with the *rrnC* terminator region, indicating that it ends within *rrnC* (Figure 5, probes 2 and 3). Similarly, the ∼400 kb fragment hybridized with the *rrnA* promoter region but not with the *rrnA* terminator region, indicating that it ends within *rrnA* (Figure 5, probes 4 and 5). These results indicate that linear DNA ending in *rrn* sequences accumulates after DNA breakage, suggesting that degradation of linear DNA is slowed down by the encounter with *rrn* sequences. In the *recA recD* context, DNA degradation reaches *rrnA*, 231 kb away from the inverted *rrnE* in 1 hr, and *rrnC*, 325 kb away, in 2 hours (Figure 4D, 4E).

**Figure 6:**
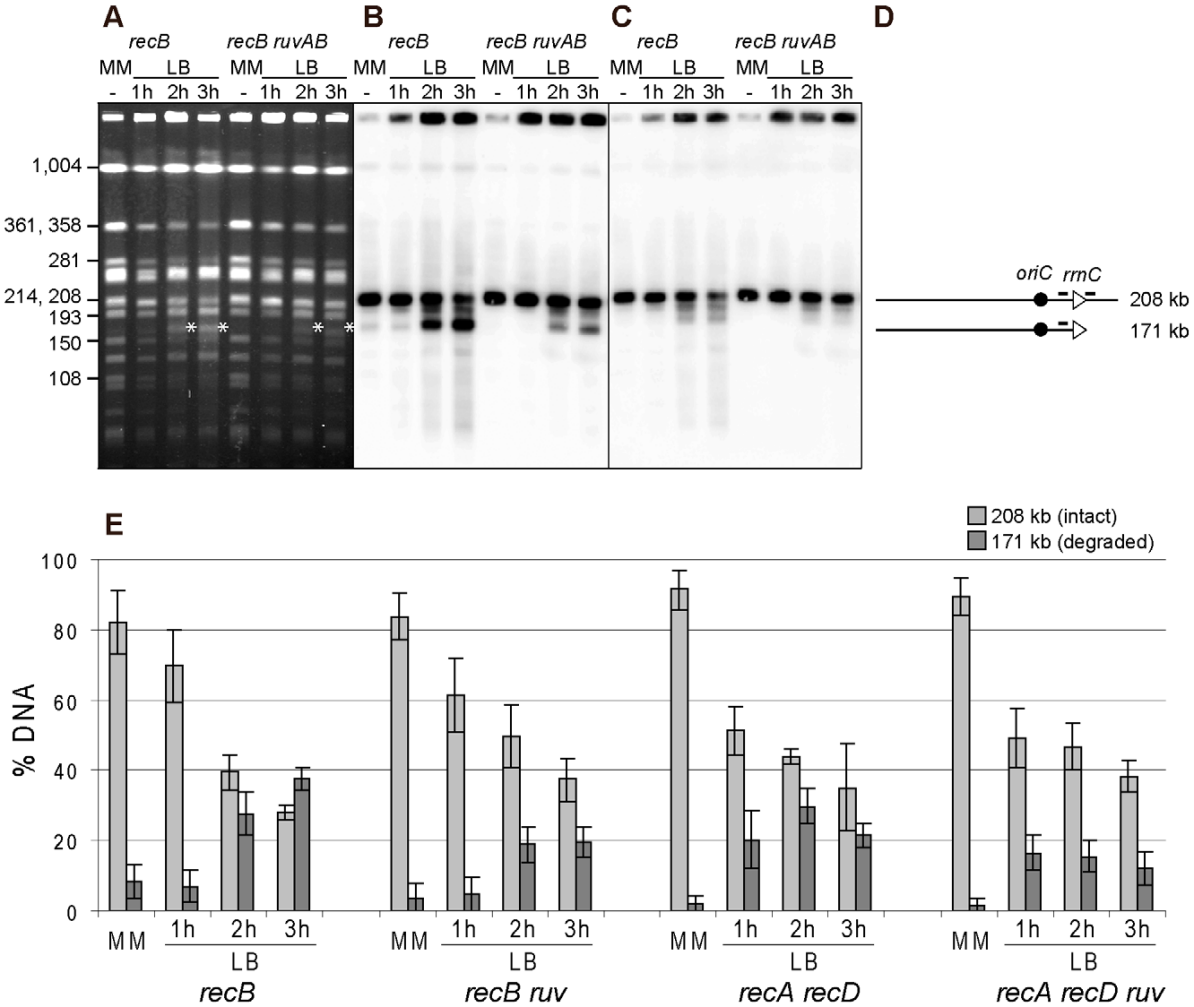
Fork breakage is partially RuvABC-dependent in InvA mutants. A. Chromosomes of the indicated InvA mutants, grown in minimal medium (MM) or for 1, 2 or 3 hours in LB, were restricted with the *Not*I enzyme and fragments were separated by PFGE. A. Ethidium bromide stained gel with *Not*I restricted chromosomes from InvA *recB* and InvA *recB ruvAB* mutants. Fragment sizes are indicated on the left. The star indicates the position of migration of the 171 kb DNA fragment formed by fork breakage and DNA degradation. B. Southern blot of the gel shown in A using the *rrnC* promoter probe, both the intact 208 kb *Not*I fragment and fragments of smaller sizes hybridize with the probe (the minor hybridization with the 193 kb *Not*1 restriction fragment may result from co-migration of broken DNA with this fragment). C. Southern blot made with the gel shown in A, using the *rrnC* terminator probe, only the intact 208 kb *Not*I fragment hybridizes with the probe. D. Schematic representation of the different DNA fragments. The triangles represent the two *rrn* operons as indicated, the black circle represents *oriC* and the bars above the lines show the positions of the probes. E. For each mutant, Southern hybridizations of 3 to 6 gels were quantified, and the percentage of hybridized DNA that remains in wells, that migrates at the 208 kb position and that migrates at the 171 kb position were calculated. For clarity, only the percentages of migrating DNA are shown here (see Table S2 for complete results); light grey, 208 kb fragment (intact), dark grey, 171 kb fragment (resulting from fork breakage and DNA degradation up to *rrnC*). Vertical bars indicate standard deviations.

In the InvA genome, *Not*I restriction produces a 208 kb fragment carrying *rrnC* (*Not*I kb 3774 to 3982, Figure 1B), and degradation of the origin distal part of this DNA fragment until *rrnC* is expected to produce a 171 kb DNA fragment (*Not*I kb 3774 to *rrnC*, Figure 1B). Actually, when the InvA *recB* mutant was grown for 2 hrs in LB, an additional ∼170 kb fragment was observed after *Not*I restriction, compared to the restriction profile of chromosomes extracted from the same cells grown in MM (Figure 6A). This DNA fragment hybridized only with a probe adjacent to the *rrnC* promoter and not with a probe adjacent to the *rrnC* terminator sequence (Figure 6B, 6C). This result indicates that the fragment ends within *rrnC*, which suggests that DNA degradation is slowed down in *rrnC* in InvA *recB* cells. This *Not*I fragment corresponds to the ∼300 kb fragment observed after cleavage by I-*Sce*I (Figure 1B), and indicates that DNA 93 kb in length, the distance between *rrnC* and the inverted *rrnA*, is degraded in 2 hours by RecJ in a *recB* context, and in 1 hour by RecJ and RecBC in a *recA recD* context (Figure 3). No 171 kb DNA fragments were observed with control strains (Figure S1).

### DNA breakage at an inverted *rrn* locus is RecA-independent and partly RuvABC-dependent

The above experiments indicate that following replication arrest at an inverted *rrnA* locus, the products of fork breakage are converted by DNA degradation, in the absence of the exonuclease V activity of RecBCD, to smaller chromosome fragments, most of which have a specific length and can be analyzed by *Not*I restriction enzyme digestion of chromosomes. As the dramatic loss of the intact band was not observed after *Not*I cleavage, in contrast to I-*Sce*I cleaved chromosomes, we used Southern blots of *Not*I fragments to quantify the linear DNA resulting from breakage at the inverted *rrnA* locus, at different times after a shift to LB and in different recombination mutants. The intensities of bands corresponding to the 208 kb *Not*I fragment (i) trapped in wells, (ii) intact (208 kb), or (iii) degraded to *rrnC* after fork breakage at InvA (171 kb), were quantified by Southern hybridization. The proportion of DNA present in each of these three bands was calculated and averages from 3 to 5 experiments are shown in Figure 6E and Table S2. In the InvA *recB* mutant, the proportion of the 171 kb DNA fragment produced by fork breakage and DNA degradation up to *rrnC* increased from 9% in MM to 27%±5%, and 37%±3%, after two and three hours in LB, respectively. In the InvA *recA recD* mutant, this proportion increased to 20%±8% after 1 hour in LB and 30%±5% after two hours, and then decreased, possibly because DNA degradation progresses beyond *rrnC*. The 171 kb DNA band appeared earlier on gels in a *recA recD* context than in a *recB* context, probably because DNA degradation is more rapid in the former mutant, owing to the combined action of RecBC and RecJ. The observation of a similar efficiency of fork breakage in InvA *recB* and in InvA *recA recD* cells confirms that fork breakage is independent of RecA. As expected, the 171 kb fragment was not observed in control strains: recombination mutants without chromosome inversion, a recombination proficient InvA mutant, and an InvA mutant in which only *recA* or only *recD* is inactivated (data not shown; Table S2).

It should be noted that, given the absence of the origin-distal dsDNA end at the replication-transcription collision site (Figure 3C, Figure 4C), the origin-proximal dsDNA end is produced by either a reaction where only one of the two replicated chromosome arms at the fork is broken, or by over-replication of blocked replication forks. Either of these reactions will produce an intact copy of the chromosome in addition to the linear DNA fragment interrupted at the position of replication-transcription collision (as in Figure 1A, step E). Therefore, the percentage of 171 kb fragments can be at most 50% of the migrating DNA, assuming 100% breakage. The percentage of chromosomes that have been replicated without DNA damage can be calculated by deducing from the measured proportion of intact 208 kb fragment that of broken DNA (171 kb fragment). These calculations show that most of the DNA is broken upon collision of replication forks with the inverted *rrn*, in a *recB* and in a *recA recD* context (Table S2).

The RFR model predicts that fork cleavage may be RuvABC dependent (Figure 1A, step E). Since RuvABC does not have any known exonuclease activity, and is not suspected to affect the activity of *E. coli* exonucleases, we used this assay to compare fork breakage in RuvABC^+^ and *ruvABC* mutant cells. The proportion of the 171 kb DNA fragment was nearly two-fold lower in InvA *recB ruvABC* compared to InvA *recB*, and in InvA *recA recD ruvABC* compared to InvA *recA recD* (Figure 6; Table S2). These observations suggest that two types of reactions are responsible for the generation of a broken chromosome arm at replication-transcription collision sites, a RuvABC-dependent (RFR) and a RuvABC-independent reaction. As expected from the LB sensitivity of Inv *recA recD* mutants, RFR is RecA-independent since the proportion of RuvABC-dependent breakage is similar in *recB* and *recA recD* contexts. Similarly, as expected from the LB sensitivity of Inv *recB recG* mutants, fork breakage was RecG-independent in an InvA *recB* context (Table S2).

## Discussion

In this work we analyze the action of recombination proteins at replication forks arrested by a collision with a highly-transcribed, oppositely-oriented *rrn* operon. We find that RecBC is crucial for replication across this region and that in its absence fork breakage, which is partly RuvABC-dependent, occurs. The RecBC requirement is caused by collisions of replication with RNA polymerases within the inverted ribosomal operon, and does not involve R-loop formation. Furthermore, in the course of this work we observed that DNA degradation of linear DNA is also arrested by an oppositely-oriented *rrn* locus.

### RFR occurs at forks arrested by a highly expressed, oppositely oriented *rrn* operon

**Figure 7:**
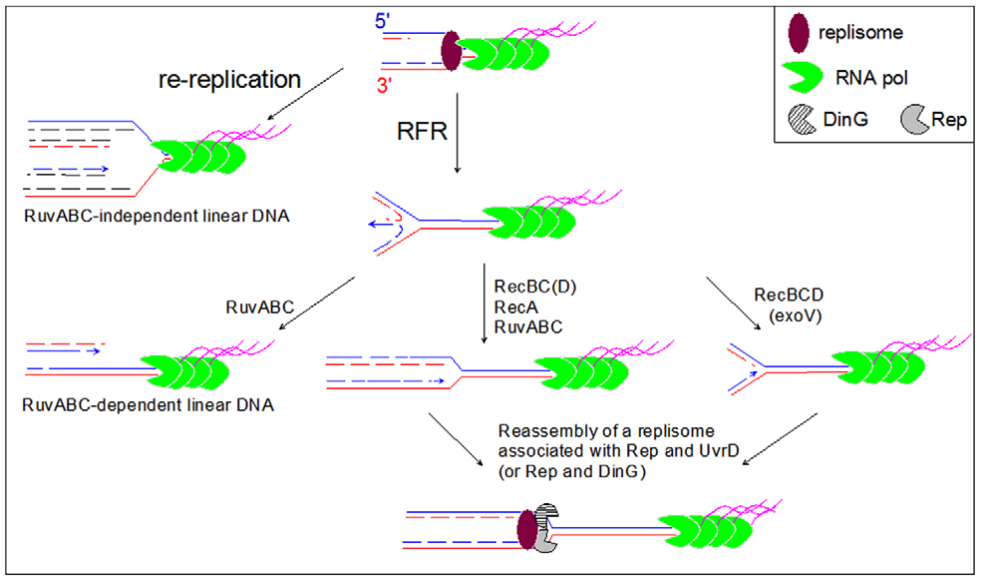
Model for the restart of forks arrested by a highly expressed, oppositely oriented *rrn* operon. A blocked fork is either reversed (RFR) or re-replicated by a following round of replication initiated at the replication origin (re-replication). The product of RuvABC-catalyzed resolution of the HJ formed by fork reversal, and the product of re-replication are similar origin-proximal dsDNA ends (left part of the model). These DNA ends are repaired in Rec^+^ cells by homologous recombination catalyzed by RecBCD, RecA and RuvABC (not shown), but remain unrepaired in *recBC* and *recA recD* mutants, where they are detected by electrophoresis of *Not*1- or I-*Sce*1-treated DNA (Figure 3, Figure 4, Figure 5, Figure 6). In Rec^+^ cells the dsDNA end formed by fork reversal can be directly acted upon by RecBCD (see Figure 1) and processed by either homologous recombination (RecBC(D)-RecA-RuvABC pathway) or by DNA degradation (RecBCD (exo V) pathway). Reversed forks resetting by either pathway produces a replication fork that has moved backward, further from the obstacle than the original blocked fork. We propose that the reloading of new replisome at such forks favors the binding of a second accessory helicase (DinG or UvrD), required with Rep for replication across the inverted *rrn* operon.

Several lines of evidence argue for the occurrence of RFR upon collision of replication with inverted *rrn* operons. First, Inv *recB* and Inv *recA recD* mutants are sensitive to growth in rich medium, whereas Inv *recA* and Inv *recD* mutants are not. Second, the inverted *rrn* genes are sites of chromosome breakage, half of which is dependent of RuvABC. We conclude that half of the linear DNA observed in Inv *recB* cells results from the resolution of reversed forks by RuvABC while the other half results from one or more other processes (Figure 7). We hypothesize that the linear DNA observed in *recB ruvAB* mutants may result from re-initiation at the chromosome origin *oriC*. Running of these new replication forks into blocked forks is expected to form linear DNA by copying the newly-synthesized strands of the first forks to the end (Figure 7). Such a run-off reaction has previously been observed at extra Ter sites inserted in the chromosome, and in over-initiation mutants [44], [45]. Strains carrying extra Ter sites are only viable if the dsDNA end is repaired by homologous recombination, suggesting that Inv cells that suffer re-replication should also require homologous recombination for replication restart and viability. Re-replication in Inv mutants may not frequent enough to render RecA essential for growth, probably owing to a significant proportion of blocked forks processed by RFR (Figure 7). Replication re-initiation at *oriC* might explain the high level of trapped I-*Sce*I fragments in LB, a RecA-independent phenomenon that was less important with the smaller *Not*I *oriC*-carrying fragment.

### DNA degradation is slowed down by the encounter of *rrn* operons

We observed that the dsDNA fragments detected in PFGE are subjected to DNA degradation by RecJ, and this reaction is more efficient in the presence of the RecBC helicase. Degradation of dsDNA ends and homologous recombination catalyzed by the combined action of RecBC and various exonucleases including RecJ, have been reported previously in a *recD* mutant [46], [47]. The reaction observed here is slow, on average around 3 kb per minute in a *recA recD* context where both RecJ and RecBC are active, and around 1 kb per minute in a *recB* mutant where it depends primarily on RecJ. RecJ is a 5′ to 3′ ssDNA exonuclease, but it can also digest dsDNA *in vitro*, a reaction that is stimulated in the presence of the RecQ helicase [48]. RecBCD digests dsDNA at a rate close to 1 kb per second [49], which allows it to rescue reversed forks prior to the resolution of the HJ (Figure 1A step D). In contrast, the genetic properties of strains that undergo RFR indicate that RecJ and RecBC do not to rescue reversed forks by DNA degradation. Actually, RecJ digests 5′ ended ssDNA at a rate of about 1 kb per minute *in vitro* [42]. A RecBCD mutant complex, in which the helicase function of the RecD subunit is inactivated, is three times slower than the intact enzyme *in vitro* [49]. This may explain why RecJ, and RecBC in the absence of RecD, do not catch up with the RuvAB-migrated HJ prior to resolution by RuvC. In addition RecJ and RecBC are less processive than RecBCD [42], [49] and the dsDNA degradation that we observe in this work is likely to result from multiple DNA binding events. Why this DNA degradation slows down at ribosomal operons remains an open question. Some features of the *rrn* locus, either structural (the operon contains DNA sequences prone to the formation of secondary structures), or functional (the operon might be expressed, even on a broken DNA arm), might trigger the dissociation of RecJ or RecBC from DNA.

### Enzymes that catalyze replication fork reversal

We have not identified the enzymatic function(s) that reverse forks in Inv strains, as the inactivation of the enzymes previously shown to reverse forks *in vivo* or *in vitro*, RecA, RuvAB or RecG, did not prevent RFR. The Rep, UvrD, and DinG helicases, which act at blocked forks in Inv mutants, are not responsible for RFR since the inactivation of any of them aggravated rather than relieved the requirement for RecBC (our unpublished results). Similarly in a *dnaNts* mutant, impaired for the β-clamp subunit of DNA polymerase III, forks are reversed by a yet unknown function, indicating that more RFR-catalyzing enzymes remain to be identified in *E. coli* [50].

Recently, replication fork reversal was proposed to occur in cells where the Rho terminator of transcription is inactivated by a specific agent, bicyclomycin [51]. Inactivating Rho-dependent transcription termination is thought to cause replication-transcription conflicts, and bicyclomycin treatment killed *recB* mutants but did not kill *recA* mutants. In the *recB* mutants, a high level of chromosome breakage was detected, suggestive of the occurrence of RFR. Chromosome breakage was attributed to the resolution by RuvABC of reversed forks, but the role of RuvABC in bicyclomycin-induced chromosome breakage was not tested. Because the amount of linear DNA measured by PFGE was strongly decreased in a *recA* mutant, it was proposed that RecA was the enzyme responsible for fork reversal. This interpretation of the data is questionable since in *recA* cells the very low level of detectable linear chromosomes could reasonably be ascribed to their degradation by RecBCD [22], [52]. Actually, we observed here that fork breakage at replication-transcription collision sites is RecA-independent.

### RFR is caused by the encounter of replication forks with RNA polymerases

Two kinds of obstacles can impede replication progression across highly transcribed regions: collisions with R-loops, and collisions with DNA-bound, transcribing (or arrested) RNA polymerases. R-loops have been shown to cause replication arrest in several organisms and to stimulate DNA rearrangements, often associated with replication fork blockage ([53], [54] and Ref therein). However, R-loops are unlikely to be the cause of replication fork arrest and reversal in Inv *recB* mutants, because over-expression of RNaseH does not suppress the sensitivity to rich medium of these strains. DinG plays two roles in Inv mutants, as it participates to RNA Pol removal together with Rep and UvrD, and it is the helicase responsible for the removal of R-loops [8]. *In vitro* studies showed that DinG can remove R-loops by directly recognizing them, independently of the presence of a replication fork [55]. The lack of effects of RNaseH over-expression in the Inv *recB* mutants leads us to conclude that the presence of DinG actually prevents the accumulation of R-loops in these mutants, and, in turn, that forks are mostly arrested at highly expressed inverted *rrn* genes by trains of RNA polymerases transcribing the rDNA.

The occurrence of RFR at inverted *rrn* genes is in agreement with the idea that replication arrest is mainly due to collisions of replication forks with RNA polymerases in a *rep* single mutant, where RFR was first reported. However, in the *rep* mutant the conversion of forks into reversed forks was partly catalyzed by RuvAB and partly by an unidentified function [56]. Here we have no evidence for RuvAB catalyzing some of the RFR reactions, since inactivating *ruvAB* does not rescue the Inv *recB* or Inv *recA recD* mutants, possibly because the alternative function can reverse all forks at the inverted *rrn* locus in the absence of RuvAB.

### RFR precedes and may facilitate the action of accessory replicative helicases

We have shown that after replication-transcription collisions, replication restart requires the action of the accessory replicative helicases Rep, UvrD and DinG, which presumably remove RNA polymerases from DNA [8]. The occurrence of RFR indicates that these helicases do not act first, since their action would allow forks to progress unimpeded across the inverted *rrn*, preventing RFR. In other words, why would RecBC be essential for growth in the presence of Rep, UvrD and DinG, if these helicases could directly act at blocked forks to remove obstacles? Therefore, we propose that RFR takes place first and that helicases act at forks that are reset after reversal (Figure 7). We can envision different reasons why RFR would take place first: either the enzyme that promotes the reaction may have more affinity for blocked forks than the accessory replicative helicases, or there may be too many RNA polymerases on highly expressed *rrn* operons for the helicases to deal with them within the time frame that is required to initiate RFR, or RFR may be required for the action of these helicases. In support for the latter hypothesis, UvrD was previously shown to act in conjunction with RecBC in two cases of replication fork restart. Firstly, UvrD acts together with homologous recombination for the removal of Tus from extra Ter sites. UvrD is then required for viability, and does not directly remove Tus from Tus/Ter-blocked forks, since it does not bypass the need for homologous recombination [57]. Secondly, UvrD is essential for viability in the *rep* mutant, where it necessarily acts after RFR, since it does not bypass the need for RecBC. These observations suggest that UvrD might more easily find its target on PriA-dependent restarted forks formed after homologous recombination, or after degradation of reversed forks. At forks blocked by an inverted *rrn* locus, the concerted action of two accessory helicases is required for restart, which are Rep, and either UvrD or DinG [8], [32]. However, in contrast with UvrD, Rep acts without homologous recombination or RFR, since it acts in wild-type *E. coli* where RecBC is not essential for viability (RecBC becomes essential only in its absence, when UvrD is required). Rep may be directly targeted to blocked forks by its interaction with the DnaB helicase [33], [35]. We propose that RFR occurs at forks blocked by an inverted *rrn* locus to promote the action of UvrD or DinG, required here in addition to Rep (Figure 7). Forks that have been reversed, and then converted back to a fork structure either by homologous recombination or by degradation of the DNA double-strand end in the reversed fork may be easier targets for UvrD and DinG than the original blocked forks. Although the molecular mechanism by which fork reversal facilitates replication restart is not known at present, we can speculate that replication fork reversal may trigger the disassembly of the blocked replisome and thereby facilitates access to DNA for the accessory helicases, and/or allow the targeting of these helicases to restarting forks by some interaction with a recombination or replication restart protein.

## Materials and Methods

### Strains and constructions

Strains and plasmids used in this work are described in Table S3 (Ref. in Text S1). They were constructed by P1 transduction. The *recB* mutation was introduced in the presence of the wild-type *recBCD* genes carried on an IPTG-dependent plasmid pAM-RecBCD. A pAM-RecA plasmid was used for propagation and P1 transduction of *recA* mutants. Plasmids were cured prior to each experiment by growing cells in the absence of IPTG and plasmid-less colonies were isolated on minimal medium (MM). All other mutations were directly introduced in the Inv mutants by P1 transduction on MM. For insertion of the I-*Sce*1 site into the chromosome, the following double-stranded sequence 5′ GCATGC*TAGGGATAACAGGGTAAT*ATCGAT 3′ carrying the I-*Sce*1 cleavage site (in italics) was inserted between the *Cla*1 and *Sph*1 sites of plasmid pKD3 [58]. Then the site was amplified by PCR together with the adjacent FRT-Cm^R^-FRT region of pKD3 and the PCR product was inserted into the chromosome as described [58].

### Measures of viability

Appropriate dilutions of overnight cultures in MM were plated on MM and LB plates. Plates were incubated at 37°C and the number of colony forming units was counted after 16–24 hours of incubation for LB plates and after 48 hours of incubation for MM plates.

### Fork breakage analysis

For each experiment, freshly isolated colonies of *recB* or *recA recD* mutants cured of the pAM-RecBCD plasmid were used for over-night cultures. However, for some mutants that grow slowly in MM, overnight cultures of cells carrying pAM-RecBCD were grown in the absence of IPTG. These cultures were plated on plates with or without Ap and IPTG and they contained less than 5% of plasmid carrying cells. This protocol was used for the following mutants: InvBE and InvA *recB recJ*, InvBE and InvA *recA recD ruvAB*, and InvA *recB ruvAB*. Plugs preparation, *Not*1 restriction, and PFGE of *Not*1 digested DNA were performed as described in [8]. Cleavage of DNA by I-*Sce*1 was performed in plugs as recommended by the supplier, with 1 hr incubation at 37°C in the presence of 2.5 units of enzyme. PFGE of I-*Sce*1 restricted chromosome was as for *Not*1, but with a ramp of pulses from 40 s to 70 s and migration for 18 hours. Transfer, hybridization, probe preparation, and quantification with Image Quant were performed as described in [8]. In Figure 3 and Figure 4 the origin proximal probe is in the *slp* gene and the origin distal probe in the *cycA* gene. Probes used in Figure 5 are in the following genes: probe 1 - origin-proximal probe: *slp* gene, probe 2 - *rrnC* promoter probe: *yieP* gene, probe 3 - *rrnC* terminator probe: *hdfR* gene, probe 4 - *rrnA* promoter probe: *hemG* gene, probe 5 - *rrnA* terminator probe: *mobA-yihE* genes, probe 6 - origin-distal probe: *cycA* gene. Probes used in Figure 6 are *rrnC* promoter probe: *yieP* gene, and *rrnC* terminator probe: *hdfR* gene.

## Supporting Information

Figure S1 Control strains do not produce linear DNA when shifted to rich medium. For all experiments shown in Figure 3, Figure 4, Figure 5, Figure 6, plugs of a control strain expected to show no fork breakage (a non-inverted *recB* or *recA recD* mutant, a InvA or InvBE RecBC^+^ mutant) were prepared in parallel with plugs of Inv *recBC* or Inv *recA recD* mutants. Each PFGE gel, and consequently each membrane used for Southern blotting, carried at least one such control, that indeed showed no fork breakage, as shown here. Representative examples of these control lanes are shown here. Top panels I-Sce1-treated chromosomes from: A - Non-inverted strains, Rec+ (wt, JJC5823), *recB* (JJC5826) and *recA recD* (JJC5912 cured of pAM-RecA+); B - InvA (JJC5891). C - InvBE *recJ* (JJC5852), InvBE *recD* (JJC5898) and InvBE *recA* (JJC5911 cured of pAM-RecA+). Bottom panels *Not*1-treated chromosomes from: D – non inverted strains, *recB* (JJC5826) and *recA recD* (JJC5912 cured of pAM-RecA+); E – InvA (JJC4010). Some DNA fragments of small size could reproducibly be detected in the non-inverted *recA recD* mutant (panel A left lanes), suggesting spontaneous breakage in this mutant. This linear DNA amounts to 5–10% at most of the total DNA in the lane, far below that produced in inverted strains, 60–70% in average in the InvA *recA recD* mutant (see Figure 3D and 3E) and 25–45% in average in the InvBE *recA recD* mutant (see Figure 4D and 4E).
(TIF)

Table S1 Inactivation of the *mfd* gene does not affect the viability of Inv mutants.
(DOC)

Table S2 Quantification of fork breakage.
(DOC)

Table S3 Strains and plasmids.
(DOC)

Text S1 Supplementary references.
(DOC)
