# Supplementary figures and images for: Replication Fork Reversal after Replication–Transcription Collision
Source: PLoS Genet. 2012 Apr 5;8(4):e1002622. doi: 10.1371/journal.pgen.1002622 (PMC3320595; doi:10.1371/journal.pgen.1002622)

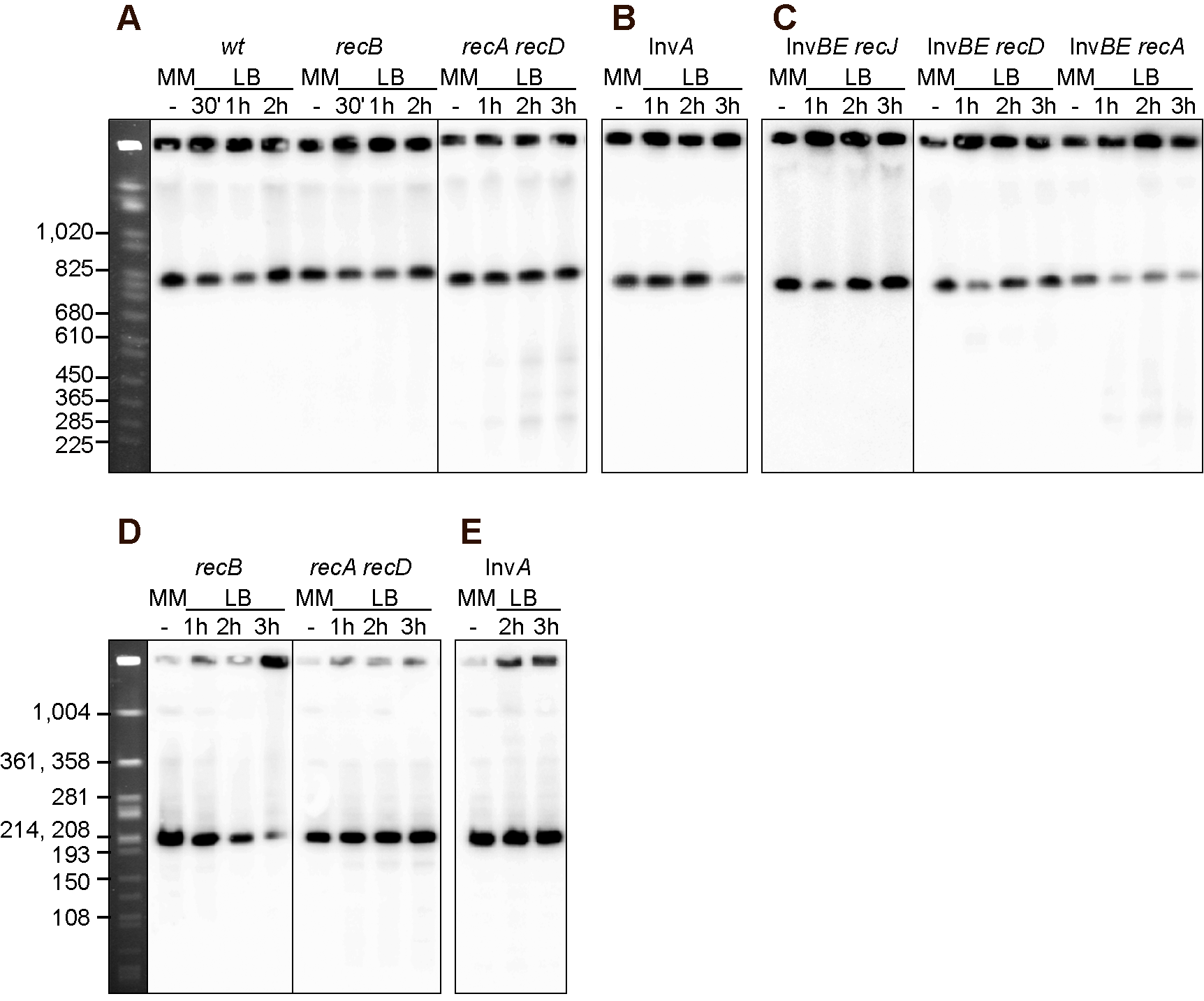

Supplement: Figure S1 — Control strains do not produce linear DNA when shifted to rich medium. For all experiments shown in Figure 3, Figure 4, Figure 5, Figure 6, plugs of a control strain expected to show no fork breakage (a non-inverted recB or recA recD mutant, a InvA or InvBE RecBC+ mutant) were prepared in parallel with plugs of Inv recBC or Inv recA recD mutants. Each PFGE gel, and consequently each membrane used for Southern blotting, carried at least one such control, that indeed showed no fork breakage, as shown here. Representative examples of these control lanes are shown here. Top panels I-Sce1-treated chromosomes from: A - Non-inverted strains, Rec+ (wt, JJC5823), recB (JJC5826) and recA recD (JJC5912 cured of pAM-RecA+); B - InvA (JJC5891). C - InvBE recJ (JJC5852), InvBE recD (JJC5898) and InvBE recA (JJC5911 cured of pAM-RecA+). Bottom panels Not1-treated chromosomes from: D – non inverted strains, recB (JJC5826) and recA recD (JJC5912 cured of pAM-RecA+); E – InvA (JJC4010). Some DNA fragments of small size could reproducibly be detected in the non-inverted recA recD mutant (panel A left lanes), suggesting spontaneous breakage in this mutant. This linear DNA amounts to 5–10% at most of the total DNA in the lane, far below that produced in inverted strains, 60–70% in average in the InvA recA recD mutant (see Figure 3D and 3E) and 25–45% in average in the InvBE recA recD mutant (see Figure 4D and 4E). (TIF) [file pgen.1002622.s001.tif]
