# Supplementary material for: Replication Fork Reversal after Replication–Transcription Collision
Source: PLoS Genet. 2012 Apr 5;8(4):e1002622. doi: 10.1371/journal.pgen.1002622 (PMC3320595; doi:10.1371/journal.pgen.1002622)
Supplement: Table S1 — Inactivation of the mfd gene does not affect the viability of Inv mutants. (DOC) [file pgen.1002622.s002.doc]

**Replication fork reversal after replication-transcription collisions.**

**De Septenville A., Duigou S^1^., Boubakri H^1^. and Michel B.**

**Table S1 Mfd does not affect the viability of strains that carry an inverted *rrn* operon**.

| strain | genotype | MM | LB |
| --- | --- | --- | --- |
| JJC4315/4349 | InvBE | 1.2 x 10^9^ ± 4.6 x 10^8^ | 1.1 x 10^9^ ± 4.3 x 10^8^ |
| JJC6075/6079 | InvBE Δ*mfd* | 1.2 x 10^9^ ± 1.6 x 10^8^ | 1.1 x 10^9^ ± 3.7 x 10^8^ |
| JJC4700S/4978S | InvBE Δ*rep* | 1.3 x 10^9^ ± 5.6 x 10^8^ | 1 x 10^9^ ± 8.4 x 10^8^  small colonies |
| JJC6086S* | InvBE Δ*rep* Δ*mfd* | 1.2 x 10^9^ ± 4 x 10^8^ | 1.1 x 10^9^ ± 3.4 x 10^8^  small colonies |
| JJC4870/4997 | InvBE Δ*uvrD* | 9.6 x 10^8^ ± 2.8 x 10^8^ | 9.3 x 10^8^ ± 3.1 x 10^8^ |
| JJC6084 | InvBE Δ*uvrD* Δ*mfd* | 1.2 x 10^9^ ± 1.4 x 10^8^ | 1.2 x 10^9^ ± 5 x 10^8^ |
| JJC4920 | InvBE Δ*dinG* | 1.2 x 10^9^ ± 5.1 x 10^8^ | *4 x 10^8^ ± 2.8 x 10^8^* |
| JJC6085 | InvBE Δ*dinG* Δ*mfd* | 1.2 x 10^9^ ± 3 x 10^8^ | *5.2 x 10^8^ ± 2.8 x 10^8^* |

S: the pAM-rep plasmid used for strain construction is cured prior to the experiment

Appropriate dilutions of over-night cultures grown in MM at 37°C were plated on MM and LB plates and colonies were counted after 1 day (LB) or 2 days (MM) of incubation at 37°C, except for InvBE Δ*dinG* and InvBE Δ*dinG* Δ*mfd* colonies that appear in two days on LB (in italics) as on MM [1]. Results for Mfd^+^ cells are the average of results published in [1] and new data obtained in parallel with Δ*mfd* cells, with the exception of the InvBE strain that was not re-tested. As shown here, the inactivation of *mfd* did not affect the plating efficiency on MM medium (low level of *rrn* expression) or on rich medium (high level of *rrn* expression) of any of the tested Inv mutant strains. We conclude that the transcription-coupled repair factor Mfd does not play a detectable role in the viability of Inv mutants, although this helicase dislodges transcription complexes blocked *in vivo* by a DNA lesion and *in vitro* by various obstacles, including replication forks [2,3,4]. Briefly, we can envision at least four reasons why Mfd does not act at inverted *rrn*: 1) it is possible that only proteins that are either targeted to replication forks or abundant can act at blocked replication forks and Mfd has never been shown to interact with a replication fork component (in contrast for example with Rep which interacts with DnaB, [5]) and is not known to be overproduced in the condition of replication blockage (in contrast with UvrD and DinG which need to be SOS-induced to act in Inv mutants, [1]). 2) Mfd interacts with RNA polymerase and acts on backtracked RNA polymerases by pushing them forward [3], blocked RNA polymerases may not backtrack in our system, or may not adopt any other conformation required for their recognition by Mfd. 3) Mfd may be unable to recognize or to act upon RNA polymerases that are modified by the anti-termination system, active in all *rrn* operons. 4) On highly-expressed *rrn* the number of RNA polymerases working at the same time may be too high for the capacity of action of Mfd. However, there is a lower density of RNA polymerases per *rrn* in MM and the viability of InvBE on MM was not dependent on Mfd activity (all mutants presented here could be constructed on MM with the expected efficiency).
