# Supplementary material for: Replication Fork Reversal after Replication–Transcription Collision
Source: PLoS Genet. 2012 Apr 5;8(4):e1002622. doi: 10.1371/journal.pgen.1002622 (PMC3320595; doi:10.1371/journal.pgen.1002622)
Supplement: Table S2 — Quantification of fork breakage. (DOC) [file pgen.1002622.s003.doc]

**Replication fork reversal after replication-transcription collisions.**

**De Septenville A., Duigou S^1^., Boubakri H^1^. and Michel B.**

**Table S2. Measures of the percentages of intact, broken and non-migrating 208 kb NotI fragment in different InvA recombination mutants show that fork breakage is RecA-independent and partially RuvAB-dependent.**

| Genotype  InvA mutants | Measured 208 kb fragment (intact) (%) | | | | Measured 171 kb fragment (broken) (%) | | | | Calculated 208 kb fragment  (% real intact)^a^ | | | | Measured non-migrating DNA (%) | | | |
| --- | --- | --- | --- | --- | --- | --- | --- | --- | --- | --- | --- | --- | --- | --- | --- | --- |
|  | MM | LB  1h | LB 2h | LB 3h | MM | LB 1h | LB 2h | LB 3h | MM | LB 1h | LB 2h | LB 3h | MM | LB  1h | LB  2h | LB  3h |
| *recB* | 82±9 | 68±10 | 40±4 | 29±3 | 9±5 | 7±4 | 27±5 | 37±3 | 73 | 61 | 13 | 0 | 9±4 | 25±6 | 33±7 | 33±4 |
| *recB ruv* | 84±7 | 61±11 | 50±9 | 37±6 | 4±4 | 5±4 | 19±5 | 20±4 | 80 | 56 | 31 | 17 | 12±3 | 34±9 | 31±9 | 43±2 |
| *recB recG* | 80±6 | 57±10 | 38±8 | 37±5 | 2±1 | 4±4 | 29±4 | 37±5 | 78 | 53 | 9 | 0 | 18±6 | 39±12 | 32±10 | 26±5 |
| *recA recD* | 91±6 | 51±7 | 44±2 | 35±12 | 2±2 | 20±8 | 30±5 | 21±4 | 89 | 31 | 14 | 14 | 6±4 | 29±6 | 26±5 | 43±11 |
| *recA recD ruv* | 89±5 | 49±8 | 47±7 | 38±4 | 2±2 | 16±5 | 16±4 | 12±5 | 88 | 33 | 31 | 26 | 9±4 | 34±4 | 38±5 | 50±6 |
| *recA* | 85±14 | 71±2 | 76±7 | 73±1 | 2±1 | 4±2 | 4±1 | 4±2 | 83 | 67 | 72 | 69 | 13±11 | 25±4 | 20±6 | 23±3 |
| *recD* | 91±6 | 70±2 | 87±5 | 90±2 | 4±4 | 3±4 | 3±2 | 2±1 | 87 | 68 | 84 | 88 | 5±2 | 27±1 | 10±2 | 8±2 |
