# Supplementary material for: Replication Fork Reversal after Replication–Transcription Collision
Source: PLoS Genet. 2012 Apr 5;8(4):e1002622. doi: 10.1371/journal.pgen.1002622 (PMC3320595; doi:10.1371/journal.pgen.1002622)
Supplement: Table S3 — Strains and plasmids. (DOC) [file pgen.1002622.s004.doc]

**Replication fork reversal after replication-transcription collisions.**

**De Septenville A., Duigou S^1^., Boubakri H^1^. and Michel B.**

**Table S3 Strains and plasmids**

| Strain | Relevant genotype | Construction or reference |
| --- | --- | --- |
| JJC3523 | MG1655 Δ*lacZ* | [6] |
| JJC3524 | MG1655 Δ*lacZ* Δ*attB* ::spcR | MG1657 in [6] |
| JJC3940 | Δ*recBCD*::Cm^R^ [pGB-recBCD+] | SS5467 (provided by Dr. S. Sandler, Amherst University, USA) transformed with pGB2-RecBCD+. |
| JJC4629 | *attL*2 *attR*45-kan^R^ Δ*recA*::cmR | [1] |
| JJC5073 | *attL*2 *attR*45-kan^R^ Δ*rrnE* | [1] |
| JJC5231 | Δ*mfd*:: Cm^R^ | DY330 in which *the mfd* gene has been entirely deleted and replaced by pKD3 Cm^R^ gene [7] |
| JJC5357 | Δ*recG*:: Cm^R^ | JJC3523 * P1 N4452 (provided by R.G. Lloyd, Nottingham University, UK) |
| JJC5384 | Δ*recBCD*::Cm^R^ [pAM-recBCD+] | JJC3523 * P1 3940 |
| JJC5385 | *recB268*::Tn*10* [pAM-recBCD+] | JJC3523 * P1 JJC777 (a *recB268*::Tn*10* mutant harbouring pDWS2 = pBR322-RecBCD+). |
| JJC5386 | Δ*recBCD*::Cm^R^ *ruvA60*::Tn*10* [pAM-recBCD+] | JJC3523-*ruvA60*::Tn*10* [pAM-recBCD+] * P1 SS5467 |
| JJC5387 | *recB268*::Tn*10*  Δ*ruvC*:: Cm^R^ [pAM-recBCD+] | JJC3523-Δ*ruvC*::Cm^R^ [pAM-recBCD+] * P1 JJC777 |
| JJC5388 | Δ*recBCD*::Cm^R^ *recJ*::Tn*10* [pAM-recBCD+] | JJC3523-*recJ*::Tn*10* [pAM-recBCD+] * P1 SS5467 |
| JJC5389 | *recB268*::Tn*10* Δ*ruvA100*:: Cm^R^ [pAM-recBCD+] | JJC3523-Δ*ruvA100*:: Cm^R^ [pAM-recBCD+] * P1 JJC777 |
| JJC5390 | *recB268*::Tn*10* Δ*recG*:: Cm^R^ [pAM-recBCD+] | JJC5357 [pAM-recBCD+] * P1 JJC777 |
| JJC5427 | *recB268*::Tn*10* Δ*ruvC*:: Cm^R^ [pAM-recBCD+] | JJC5385 * P1 Δ*ruvC*::Cm^R^ (from JJC783, laboratory collection) |
| JJC5428 | *recB268*::Tn*10* Δ*recG*:: Cm^R^ [pAM-recBCD+] | JJC5385 * P1 N4452 |
| JJC5429 | Δ*recBCD*::Cm^R^ *recJ*::Tn*10* [pAM-recBCD+] | JJC5384 * P1 *recJ*::Tn*10* (from JJC104, laboratory collection) |
| JJC5484 | Δ*recA*::Cm^R^ *recD*::Tn*10* [pAM-recA+] | JJC3523-Δ*recA*:: Cm^R^ [pAM-recA+] * P1 *recD*::Tn*10* (from JJC276, laboratory collection) |
| JJC5507 | Δ*ruvAB*::Cm^R^ | JJC3524*P1 DY330 in which the *ruvAB* operon has been deleted from the 28^th^ nucleotide of *ruvA* to the 962^th^ nucleotide of *ruvB* and replaced by pKD3 Cm^R^ gene [7] |
| JJC5530 | Δ*ruvAB*::FRT | JJC5507 excised of the Cm^R^ gene by FRT activation using pCP20 [7] |
| JJC5548 | Δ*ruvAB*::FRT Δ*recA*::Cm^R^ | JJC5530 * P1 Δ*recA*::Cm^R^ (from JJC354, laboratory collection) |
| JJC5553 | Δ*recBD*::Cm^R^ | DY330 in which *recD* and *recB* adjacent genes have been deleted from the 24^th^ nucleotide of *recD* to the 2829^th^ nucleotide of *recB* and replaced by pKD3 Cm^R^ gene [7] |
| JJC5554 | Δ*recBD*::Cm^R^ [pAM-recBCD+] | JJC5553 + [pAM-recBCD+] |
| JJC5563 | Δ*ruvAB*::FRT Δ*recA*::Cm^R^ [pAM-recA+] | JJC5548 + [pAM-recA+] |
| JJC5568 | Δ*recBD*::FRT | JJC3523 * P1 JJC5554 and excised of the Cm^R^ gene by FRT activation using pCP20 [7] |
| JJC5571 | Δ*ruvAB*::FRT Δ*recA*::Cm^R^ *recD*::Tn*10* [pAM-recA+] | JJC5563* P1 *recD*::Tn*10* (from JJC276, laboratory collection) |
| JJC5637 | Δ*recBD*::FRT [pAM-recBCD+] | JJC5568 + [pAM-recBCD+] |
| JJC5643 | Δ*recBD*::FRT Δ*recG* ::Kan^R^ [pAM-recBCD+] | JJC5637* P1 N4452 |
| JJC5645 | Δ*recBD*::FRT Δ*recG* ::Kan^R^ [pAM-recBCD+] | JJC5637* P1 N4452 |
| JJC5788 | Δ*ruvAB*::FRT Δ*recA*::Cm^R^ [pAM-recA+] | JJC5530*P1 Δ*recA*::Cm^R^ (from JJC354, laboratory collection) + pAM-recA+ |
| JJC5790 | *ytfJ*::I-SceI Cm^R^ | DY330 in which the *ytfJ* gene has been deleted from the 363^th^ to the 428^th^ nucleotide and replaced by an I-SceI restriction site and pKD3 Cm^R^ gene [7] |
| JJC5791 | *dtpB*::I-SceI Cm^R^ | DY330 in which the *dtpB* gene has been deleted from the 294^th^ to the 346^th^ nucleotide and replaced by I-*Sce*I restriction site and the pKD3 Cm^R^ gene [7] |
| JJC5793 | *ytfJ*::I-SceI Cm^R^ | JJC3523 * P1 JJC5790 |
| JJC5794 | *dtpB*::I-SceI Cm^R^ | JJC3523 * P1 JJC5791 |
| JJC5799 | *ytfJ*::I-SceI:: FRT | JJC5793 excised of the Cm^R^ gene by FRT activation using pCP20 [7] |
| JJC5800 | *dtpB*::I-SceI:: FRT | JJC5794 excised of the Cm^R^ gene by FRT activation using pCP20 [7] |
| JJC5801 | *ytfJ*::I-SceI:: FRT *dtpB*::I-SceI:: Cm^R^ | JJC5799* P1 DY330 Δ*dtpB*::I-SceI CmR |
| JJC5823 | *ytfJ*::I-SceI::FRT *dtpB*::I-SceI::FRT | JJC5801 excised of the Cm^R^ gene by FRT activation using pCP20 [7] |
| JJC5826 | *ytfJ*::I-SceI::FRT *dtpB*::I-SceI::FRT Δ*recBD*::Cm^R^ | JJC5823*P1 JJC5554 |
| JJC5835 | *ytfJ*::I-SceI::FRT *dtpB*::I-SceI::FRT Δ*recBD*::Cm^R^ [pAM-recBCD+] | JJC5826 + [pAM-recBCD+] |
| JJC5837 | *ytfJ*::I-SceI::FRT *dtpB*::I-SceI::FRT *recJ*::Tn*10* | JJC5823*P1 *recJ*::Tn*10* (from JJC104, laboratory collection) |
| JJC5847 | *recD*::Tn*10* Δ*ruvC*:: Cm^R^ | 3524-*recD*::Tn*10* *P1 Δ*ruvC*:: Cm^R^ (from JJC783, laboratory collection) |
| JJC5838 | *ytfJ*::I-SceI::FRT *dtpB*::I-SceI::FRT Δ*recBD*::FRT | JJC5826 excised of the Cm^R^ gene by FRT activation using pCP20 [7] |
| JJC5855 | *ytfJ*::I-SceI::FRT *dtpB*::I-SceI::FRT Δ*recBD*::FRT Δ*ruvAB*::Cm^R^ [pAM-recBCD+] | JJC5838 [pAM-recBCD+] * P1 JJC5507 |
| JJC5904 | *ytfJ*::I-SceI::FRT *dtpB*::I-SceI::FRT Δ*recA*::Cm^R^ | JJC5823 * P1 Δ*recA*::Cm^R^ (from JJC354, laboratory collection) |
| JJC5907 | *ytfJ*::I-SceI::FRT *dtpB*::I-SceI::FRT Δ*recA*::Cm^R^ [pAM-recA+] | JJC5904 + [pAM-recA+] |
| JJC5912 | *ytfJ*::I-SceI::FRT *dtpB*::I-SceI::FRT Δ*recA*::Cm^R^ *recD*::Tn*10* [pAM-recA+] | JJC5907* P1 *recD*::Tn*10* (from JJC276, laboratory collection) |
| JJC5913 | *ytfJ*::I-SceI::FRT *dtpB*::I-SceI::FRT Δ*ruvAB*::FRT Δ*recA*::Cm^R^ | JJC5823-Δ*ruvAB* * P1 Δ*recA*::Cm^R^ (from JJC354, laboratory collection) |
| JJC5921 | *ytfJ*::I-SceI::FRT *dtpB*::I-SceI::FRT Δ*ruvAB*::FRT Δ*recA*::Cm^R^ [pAM-recA+] | JJC5913 + [pAM-recA+] |
| JJC5924 | *ytfJ*::I-SceI::FRT *dtpB*::I-SceI::FRT Δ*ruvAB*::FRT Δ*recA*::Cm^R^ *recD*::Tn*10* [pAM-recA+] | JJC5921* P1 *recD*::Tn*10* (from JJC276, laboratory collection) |
|  |  |  |
| **InvA strains** | | |
| JJC4010 | Inv (*attL*15-cm^R^ *attR*75-kan^R^) / InvA | [1] |
| JJC4027 | InvA Δ(*recA-srl*) ::Tn*10* [pAM-recA] | [1] |
| JJC4028 | InvA *recB268*::Tn*10* [pAM-recBCD+] | JJC4010 [pAM-recBCD+] * P1 JJC777 |
| JJC5434 | InvA Δ*recBCD*::Cm^R^ *ruvA60*::Tn*10* [pAM-recBCD+] | JJC5386 * P1 JJC4010 |
| JJC5435 | InvA *recB268*::Tn*10* Δ*ruvA100*:: Cm^R^ [pAM-recBCD+] | JJC5389 * P1 JJC4010 |
| JJC5436 | InvA *recB268*::Tn*10*  Δ*ruvC*:: Cm^R^ [pAM-recBCD+] | JJC5387 * P1 JJC4010 |
| JJC5437 | InvA *recB268*::Tn*10* Δ*recG*:: Cm^R^ [pAM-recBCD+] | JJC5390 * P1 JJC4010 |
| JJC5438 | InvA Δ*recBCD*::Cm^R^ *recJ*::Tn*10* [pAM-recBCD+] | JJC5388 * P1 JJC4010 |
| JJC5555 | InvA Δ*recA*::Cm^R^ *recD*::Tn*10* [pAM-recA+] | JJC5484 * P1 JJC4010 |
| JJC5631 | InvA Δ*ruvAB*::FRT Δ*recA*::Cm^R^ *recD*::Tn*10* [pAM-recA+] | JJC5571 * P1 JJC4010 |
| JJC5638 | InvA Δ*recG*:: Cm^R^ | JJC5357 * P1 JJC4010 |
| JJC5642 | InvA Δ*recG*:: Cm^R^ *ruvA60*::Tn*10* | JJC5638 * P1 *ruvA60*::Tn*10* (from JJC671, laboratory collection) |
| JJC5663 | InvA Δ*recBD*::FRT Δ*recG* ::Kan^R^ [pAM-recBCD+] | JJC5643 * P1 JJC4010 |
| JJC5664 | InvA Δ*recBD*::FRT Δ*recG* ::Kan^R^ [pAM-recBCD+] | JJC5645 * P1 JJC4010 |
| JJC5792 | InvA Δ*ruvAB*::FRT Δ*recA*::Cm^R^ [pAM-recA+] | JJC5788 * P1 JJC4010 |
| JJC5850 | InvA *ytfJ*::I-SceI::FRT *dtpB*::I-SceI::FRT *recJ*::Tn*10* | JJC5837 * P1 JJC4010 |
| JJC5851 | InvA *ytfJ*::I-SceI::FRT *dtpB*::I-SceI::FRT Δ*recBD*::Cm^R^ [pAM-recBCD+] | JJC5835 * P1 JJC4010 |
| JJC5859 | InvA *ytfJ*::I-SceI::FRT *dtpB*::I-SceI::FRT Δ*recBD*::FRT Δ*ruvAB*::Cm^R^ [pAM-recBCD+] | JJC5855 * P1 JJC4010 |
| JJC5860 | InvA *recD*::Tn*10* Δ*ruvC*:: Cm^R^ | JJC5847 * P1 JJC4010 |
| JJC5891 | InvA *ytfJ*::I-SceI::FRT *dtpB*::I-SceI::FRT | JJC5823 * P1 JJC4010 |
| JJC5897 | InvA *ytfJ*::I-SceI::FRT *dtpB*::I-SceI::FRT *recD*::Tn*10* | JJC5891 * P1 *recD*::Tn*10* (from JJC276, laboratory collection) |
| JJC5906 | InvA *ytfJ*::I-SceI::FRT *dtpB*::I-SceI::FRT Δ(*recA-srl*) ::Tn*10* [pAM-recA+] | JJC5891* P1 Δ*recA*::Cm^R^ (from JJC354, laboratory collection) |
| JJC5920 | InvA *ytfJ*::I-SceI::FRT *dtpB*::I-SceI::FRT Δ*recA*::Cm^R^ *recD*::Tn*10* [pAM-recA+] | JJC5912 * P1 JJC4010 |
| JJC5930 | InvA *ytfJ*::I-SceI::FRT *dtpB*::I-SceI::FRT Δ*ruvAB*::FRT Δ*recA*::Cm^R^ recD::Tn10 [pAM-recA+] | JJC5924 * P1 JJC4010 |
| JJC5974 | InvA Δ*recBD*::FRT Δ*recG* ::Kan^R^ *ruvA60*::Tn*10* [pAM-recBCD+] | JJC5663 * P1 *ruvA60*::Tn*10* (from JJC671, laboratory collection) |
| JJC5975 | InvA Δ*recBD*::FRT Δ*recG* ::Kan^R^ *ruvA60*::Tn*10* [pAM-recBCD+] | JJC5664 * P1 *ruvA60*::Tn*10* (from JJC671, laboratory collection) |
| JJC5990 | InvA *ruvA60*::Tn*10* | JJC4010 * P1 *ruvA60*::Tn*10* (from JJC671, laboratory collection) |
|  |  |  |
| **InvBE strains** | | |
| JJC4315 | Inv attL2-cm^R^ attR45-kan^R^ / InvBE | [1] |
| JJC4349 | InvBE Cm^S^ | [1] |
| JJC4384 | InvBE *recB268*::Tn*10* [pAM-recBCD+] | JJC4349 [pAM-recBCD+] * P1 JJC777 |
| JJC4394 | InvBE *recB268*::Tn*10*  Δ*ruvC*:: Cm^R^ [pAM-recBCD+] | JJC4384 * P1 Δ*ruvC*:: Cm^R^ (from JJC783, laboratory collection) |
| JJC4405 | InvBE Δ*recBCD*::Cm^R^ [pGB-recBCD+] | JJC4349* P1 JJC3940 |
| JJC4631 | InvBE Δ*recA*::Cm^R^ [pAM-recA+] | [1] |
| JJC4632 | InvBE Δ*recA*::Cm^R^ *recD*::Tn*10* [pAM-recA+] | JJC4631* P1 *recD*::Tn*10* (from JJC276, laboratory collection) |
| JJC4842 | InvBE Δ*recG*:: Cm^R^ | JJC4349 * P1 5357 |
| JJC5000 | InvBE InvBE *recB268*::Tn*10* [pAM-recBCD+] Δ*rrnE*::Cm^R^ | JJC4384 * P1 4921  Gene replacement of the *rrnE* gene by Cm^R^  Described in [1] |
| JJC5002 | InvBE Δ*recBCD*::Cm^R^ [pGB-recBCD+] *rpoC*^Δ215-220^ *thiC*::Tn*10* | JJC4405*P1 RLG3381 [8] |
| JJC5025 | InvBE Δ*ruvC*:: Cm^R^ | JJC4349* P1 Δ*ruvC*:: Cm^R^ (from JJC783, laboratory collection) |
| JJC5049 | InvBE Δ*recG*:: Cm^R^ [pAM-recBCD+] | JJC4842 + [pAM-recBCD+] |
| JJC5057 | InvBE *recB268*::Tn*10* Δ*recG*:: Cm^R^ [pAM-recBCD+] | JJC5049 * P1 JJC777 |
| JJC5263 | InvBE Δ*ruvABC*:: Cm^R^ | JJC4349 * P1 Δ*ruvABC*:: Cm^R^ (from JJC754, laboratory collection) |
| JJC5318 | InvBE *recB268*::Tn*10*  Δ*ruvABC*:: Cm^R^ | JJC4384 * P1 Δ*ruvABC*:: Cm^R^ (from JJC754, laboratory collection) cured of pAM-recBCD^+^ |
| JJC5319 | InvBE *recB268*::Tn*10*  Δ*ruvC*:: Cm^R^ [pAM-recBCD+] | JJC4384 * P1 Δ*ruvC*:: Cm^R^ (from JJC783, laboratory collection) |
| JJC5320 | InvBE *recB268*::Tn*10*  Δ*ruvABC*:: Cm^R^ [pAM-recBCD+] | JJC4384 * P1 Δ*ruvABC*:: Cm^R^ (from JJC754, laboratory collection) |
| JJC5335 | InvBE Δ(*recA-srl*) ::Tn*10* Δ*ruvC*:: Cm^R^ [pAM-recA] | JJC4349-Δ(*recA-srl*) ::Tn*10* [pAM-recA]* P1 Δ*ruvC*:: Cm^R^ (from JJC783, laboratory collection) |
| JJC5336 | InvBE Δ(*recA-srl*) ::Tn*10* Δ*ruvABC*:: Cm^R^ [pAM-recA] | JJC4349-Δ(*recA-srl*) ::Tn*10* [pAM-recA]*P1 Δ*ruvABC*:: Cm^R^ (from JJC754, laboratory collection) |
| JJC5338 | InvBE Δ*recBCD*::Cm^R^ *ruvA60*::Tn*10* [pAM-recBCD+] | JJC4405 * P1 *ruvA60*::Tn*10* (from JJC671, laboratory collection) |
| JJC5339 | InvBE Δ*recBCD*::Cm^R^ *recJ*::Tn*10* [pAM-recBCD+] | JJC4405 * P1 *recJ*::Tn*10* (from JJC104, laboratory collection) |
| JJC5368 | InvBE *recB268*::Tn*10* Δ*ruvA100*:: Cm^R^ [pAM-recBCD+] | JJC4384 * P1 Δ*ruvA100*:: Cm^R^ (from JJC3627, laboratory collection) |
| JJC5531 | InvBE Δ*ruvAB*::FRT | JJC4349 * JJC5507 and excised of the Cm^R^ gene by FRT activation using pCP20 [7] |
| JJC5573 | InvBE Δ*ruvAB*::FRT *recB268*::Tn*10*  [pAM-recBCD+] | JJC5508 * P1 JJC777 |
| JJC5639 | InvBE Δ*ruvAB*::FRT *recB268*::Tn*10*  Δ*recG*:: Cm^R^ [pAM-recBCD+] | JJC5573 * P1 N4452 |
| JJC5641 | InvBE Δ*recG*:: Cm^R^ *ruvA60*::Tn*10* | JJC5049 * P1 *ruvA60*::Tn*10* (from JJC671, laboratory collection) |
| JJC5671 | InvBE Δ*ruvAB*::FRT *recD*::Tn*10*  [pAM-recBCD+] | JJC5531 * P1 *recD*::Tn*10* (from JJC276, laboratory collection) |
| JJC5687 | InvBE Δ*ruvAB*::FRT *recD*::Tn*10*  Δ*recG*:: Cm^R^ [pAM-recBCD+] | JJC5671 * P1 N4452 |
| JJC5738 | InvBE Δ*recA*::Cm^R^ *ruvA60*::Tn*10* [pAM-recA+] | JJC4631* P1 *ruvA60*::Tn*10* (from JJC671, laboratory collection) |
| JJC5796 | InvBE *ytfJ*::I-SceI Cm^R^ | JJC4349 * P1 JJC5790 |
| JJC5807 | InvBE *ytfJ*::I-SceI::FRT | JJC5796 excised of the Cm^R^ gene by FRT activation using pCP20 [7] |
| JJC5814 | InvBE *recD*::Tn*10* | JJC4349 * P1 *ruvA60*::Tn*10* (from JJC671, laboratory collection) |
| JJC5812 | InvBE *ytfJ*::I-SceI::FRT *dtpB*::I-SceI Cm^R^ | JJC5807 * P1 JJC 5791 |
| JJC5833 | InvBE *ytfJ*::I-SceI::FRT *dtpB*::I-SceI::FRT | JJC5812 excised of the Cm^R^ gene by FRT activation using pCP20 [7] |
| JJC5843 | InvBE *recD*::Tn*10*  Δ*ruvC*:: Cm^R^ | JJC5814 * P1 Δ*ruvC*:: Cm^R^ (from JJC783, laboratory collection) |
| JJC5849 | InvBE *ytfJ*::I-SceI::FRT *dtpB*::I-SceI::FRT Δ*ruvAB*:: Cm^R^ | JJC5833 * P1 5507 |
| JJC5852 | InvBE *ytfJ*::I-SceI::FRT *dtpB*::I-SceI::FRT *recJ*::Tn*10* | JJC5833 * P1 *recJ*::Tn*10* (from JJC104, laboratory collection) |
| JJC5858 | InvBE *ytfJ*::I-SceI::FRT *dtpB*::I-SceI::FRT Δ*recBD*::Cm^R^ [pAM-recBCD+] | JJC5833 [pAM-recBCD+] * P1 JJC5554 |
| JJC5865 | InvBE *ytfJ*::I-SceI::FRT *dtpB*::I-SceI::FRT Δ*ruvAB*:: FRT | JJC5849 excised of the Cm^R^ gene by FRT activation using pCP20 [7] |
| JJC5898 | InvBE *ytfJ*::I-SceI::FRT *dtpB*::I-SceI::FRT *recD*::Tn*10* | JJC5833 * P1 *recD*::Tn*10* (from JJC276, laboratory collection) |
| JJC5911 | InvBE *ytfJ*::I-SceI::FRT *dtpB*::I-SceI::FRT Δ*recA*::Cm^R^ [pAM-recA+] | JJC5833 [pAM-recA+] * P1 Δ*recA*::Cm^R^ (from JJC354, laboratory collection) |
| JJC5918 | InvBE *ytfJ*::I-SceI::FRT *dtpB*::I-SceI::FRT Δ*recA*::Cm^R^ *recD*::Tn*10* [pAM-recA+] | JJC5911* P1 *recD*::Tn*10* (from JJC276, laboratory collection) |
| JJC5919 | InvBE *ytfJ*::I-SceI::FRT *dtpB*::I-SceI::FRT Δ*recBD*::Cm^R^ *ruvA60*::Tn*10* [pAM-recBCD+] | JJC5858 * P1 *ruvA60*::Tn*10* (from JJC671, laboratory collection) |
| JJC5923 | InvBE *ytfJ*::I-SceI::FRT *dtpB*::I-SceI::FRT Δ*ruvAB*::FRT Δ*recA*::Cm^R^ [pAM-recA+] | JJC5865 [pAM-recA+] * P1 Δ*recA*::Cm^R^ (from JJC354, laboratory collection) |
| JJC5925 | InvBE *ytfJ*::I-SceI::FRT *dtpB*::I-SceI::FRT Δ*ruvAB*::FRT Δ*recBD*::Cm^R^ [pAM-recBCD+] | JJC5865 [pAM-recBCD+] * P1 JJC5544 |
| JJC5929 | InvBE *ytfJ*::I-SceI::FRT *dtpB*::I-SceI::FRT Δ*ruvAB*::FRT Δ*recA*::Cm^R^ *recD*::Tn*10* [pAM-recA+] | JJC5923 * P1 *recD*::Tn*10* (from JJC276, laboratory collection) |
| JJC6075 | InvBE Δ*mfd*::Cm^R^ | JJC4349 * P1 JJC5231 |
| JJC6079 | InvBE Δ*mfd*::FRT | JJC6075 excised of the Cm^R^ gene by FRT activation using pCP20 [7] |
| JJC6083 | InvBE Δ*mfd*::FRT [pAM-rep+] | JJC6079 transformed by [pAM-rep+] |
| JJC6084 | InvBE Δ*mfd*::FRT Δ*uvrD*::Cm^R^ | JJC6079 * P1 JJC2457 [1] |
| JJC6085 | InvBE Δ*mfd*::FRT Δ*dinG*::Cm^R^ | JJC6079 * P1 JJC1869 [1] |
| JJC6086 | InvBE Δ*mfd*::FRT Δ*rep*::Cm^R^ [pAM-rep+] | JJC6083 * P1 JJC735 [1] |

| Plasmid Name | Reference or construction |
| --- | --- |
| pAM-RecBCD | Cloning of the RecBCD ~18 kb *Bam*H1 fragment from pDWS2 [9] in pAM-34 [10]. |
| pAM-RecA | Cloning of the SpecR gene from pAM34 [10] in pCY579 [11] |
| pEM001 | pACYC184 derivative carrying the *rnh* *E. coli* gene [12] |
| pGB-RecBCD | Cloning of the RecBCD ~18 kb *Bam*H1 fragment from pDWS2 [9] in pGB2 [13] |
| pKD3-I-Sce1 | Cloning of the double-stranded sequence 5’ GCATGC*TAGGGATAACAGGGTAAT*ATCGAT 3’ between the *Cla*1 and *Sph*1 sites of plasmid pKD3 [7] |
