# Supplementary material for: Replication Fork Reversal after Replication–Transcription Collision
Source: PLoS Genet. 2012 Apr 5;8(4):e1002622. doi: 10.1371/journal.pgen.1002622 (PMC3320595; doi:10.1371/journal.pgen.1002622)
Supplement: Text S1 — Supplementary references. (DOC) [file pgen.1002622.s005.doc]

**Replication fork reversal after replication-transcription collisions.**

**De Septenville A., Duigou S^1^., Boubakri H^1^. and Michel B.**

Text S1. Supplementary References

1. Boubakri H, de Septenville AL, Viguera E, Michel B (2010) The helicases DinG, Rep and UvrD cooperate to promote replication across transcription units in vivo. Embo J 29: 145-157.

2. Selby CP, Sancar A (1993) Molecular Mechanism of Transcription-Repair Coupling. Science 260: 53-58.

3. Park JS, Marr MT, Roberts JW (2002) E. coli Transcription repair coupling factor (Mfd protein) rescues arrested complexes by promoting forward translocation. Cell 109: 757-767.

4. Pomerantz RT, O'Donnell M (2010) Direct restart of a replication fork stalled by a head-on RNA polymerase. Science 327: 590-592.

5. Guy CP, Atkinson J, Gupta MK, Mahdi AA, Gwynn EJ, et al. (2009) Rep provides a second motor at the replisome to promote duplication of protein-bound DNA. Mol Cell 36: 654-666.

6. Valens M, Penaud S, Rossignol M, Cornet F, Boccard F (2004) Macrodomain organization of the Escherichia coli chromosome. Embo J 23: 4330-4341.

7. Datsenko KA, Wanner BL (2000) One-step inactivation of chromosomal genes in Escherichia coli K-12 using PCR products. Proc Natl Acad Sci U S A 97: 6640-6645.

8. Bartlett MS, Gaal T, Ross W, Gourse RL (1998) RNA polymerase mutants that destabilize RNA polymerase-promoter complexes alter NTP-sensing by rrn P1 promoters. J Mol Biol 279: 331-345.

9. Amundsen SK, Neiman AM, Thibodeaux SM, Smith GR (1990) Genetic dissection of the biochemical activities of RecBCD enzyme. Genetics 126: 25-40.

10. Gil D, Bouche JP (1991) ColE1-Type Vectors with Fully Repressible Replication. Gene 105: 17-22.

11. Cronan JE (2003) Cosmid-based system for transient expression and absolute off-to-on transcriptional control of Escherichia coli genes. J Bacteriol 185: 6522-6529.

12. Masse E, Phoenix P, Drolet M (1997) DNA topoisomerases regulate R-loop formation during transcription of the rrnB operon in Escherichia coli. J Biol Chem 272: 12816-12823.

13. Churchward G, Belin D, Nagamine Y (1984) A pSC101-derived plasmid which shows no sequence homology to other commonly used cloning vectors. Gene 31: 165-171.
